# Discovering Transcription Factor Binding Sites in Highly Repetitive Regions of Genomes with Multi-Read Analysis of ChIP-Seq Data

**DOI:** 10.1371/journal.pcbi.1002111

**Published:** 2011-07-14

**Authors:** Dongjun Chung, Pei Fen Kuan, Bo Li, Rajendran Sanalkumar, Kun Liang, Emery H. Bresnick, Colin Dewey, Sündüz Keleş

**Affiliations:** 1 Department of Statistics, University of Wisconsin, Madison, Wisconsin, United States of America; 2 Department of Biostatistics and Medical Informatics, University of Wisconsin, Madison, Wisconsin, United States of America; 3 Department of Biostatistics, University of North Carolina, Chapel Hill, North Carolina, United States of America; 4 Department of Computer Sciences, University of Wisconsin, Madison, Wisconsin, United States of America; 5 Wisconsin Institutes for Medical Research, UW Carbone Cancer Center, Department of Cell and Regenerative Biology, University of Wisconsin School of Medicine and Public Health, Madison, Wisconsin, United States of America; University of British Columbia, Canada

## Abstract

Chromatin immunoprecipitation followed by high-throughput sequencing (ChIP-seq) is rapidly replacing chromatin immunoprecipitation combined with genome-wide tiling array analysis (ChIP-chip) as the preferred approach for mapping transcription-factor binding sites and chromatin modifications. The state of the art for analyzing ChIP-seq data relies on using only reads that map uniquely to a relevant reference genome (uni-reads). This can lead to the omission of up to 30% of alignable reads. We describe a general approach for utilizing reads that map to multiple locations on the reference genome (multi-reads). Our approach is based on allocating multi-reads as fractional counts using a weighted alignment scheme. Using human STAT1 and mouse GATA1 ChIP-seq datasets, we illustrate that incorporation of multi-reads significantly increases sequencing depths, leads to detection of novel peaks that are not otherwise identifiable with uni-reads, and improves detection of peaks in mappable regions. We investigate various genome-wide characteristics of peaks detected only by utilization of multi-reads via computational experiments. Overall, peaks from multi-read analysis have similar characteristics to peaks that are identified by uni-reads except that the majority of them reside in segmental duplications. We further validate a number of GATA1 multi-read only peaks by independent quantitative real-time ChIP analysis and identify novel target genes of GATA1. These computational and experimental results establish that multi-reads can be of critical importance for studying transcription factor binding in highly repetitive regions of genomes with ChIP-seq experiments.

## Author Summary

Annotating repetitive regions of genomes experimentally is a challenging task. Chromatin immunoprecipitation followed by high-throughput sequencing (ChIP-seq) provides valuable data for characterizing repetitive regions of genomes in terms of transcription factor binding. Although ChIP-seq technology has been maturing, available ChIP-seq analysis methods and software rely on discarding sequence reads that map to multiple locations on the reference genome (multi-reads), thereby generating a missed opportunity for assessing transcription factor binding to highly repetitive regions of genomes. We develop a computational algorithm that takes multi-reads into account in ChIP-seq analysis. We show with computational experiments that multi-reads lead to significant increase in sequencing depths and identification of binding regions that are otherwise not identifiable when only reads that uniquely map to the reference genome (uni-reads) are used. In particular, we show that the number of binding regions identified can increase up to 36%. We support our computational predictions with independent quantitative real-time ChIP validation of binding regions identified only when multi-reads are incorporated in the analysis of a mouse GATA1 ChIP-seq experiment.

## Introduction

The introduction of next generation sequencing has enabled a myriad of creative ways to answer genome-wide questions. Chromatin immunoprecipitation coupled with high-throughput next generation sequencing (ChIP-seq) has become a powerful technique for large scale profiling of transcription factor binding and chromatin modifications [1]–[5] and is offering a powerful alternative to ChIP on microarrays (ChIP-chip) [6]–[9]. In ChIP-seq, DNA fragments are sequenced directly instead of being hybridized on an array. Although there are multiple platforms for high-throughput sequencing, the Illumina sequencer, which works by sequencing a small region (25 to 100 bp) from one or both ends of each fragment, is commonly used for ChIP-seq experiments. A ChIP-seq experiment generates millions of short *reads/tags*. The first step of data analysis is to map reads to the reference genome and retain reads that map to unique locations (*uni-reads*) [10]–[12]. Although constraining the analysis to uni-reads by discarding reads that map to multiple locations (*multi-reads*) leads to reduced coverage and sequencing depth, this may not render a serious problem in most cases. This is because many uni-reads might be adjacent to discarded multi-reads and can lead to identification of underlying peaks. However, discarding multi-reads poses a significant challenge for identifying binding locations residing in genomic regions that have been duplicated over evolutionary time since these regions will not have many uni-reads.

Shortcomings of discarding multi-reads have been recognized in transcriptome sequencing (RNA-Seq) [13]–[18]. These studies demonstrated that discarding multi-reads leads to inaccurate estimation of expression of genes that reside in repetitive regions. Gene repetitiveness may be due to either low complexity segments or recent gene duplications. Numerous studies have highlighted the biological importance of segmental duplications [19], [20]. Duplicated genes could retain their original functions or acquire new functions by changes in coding sequences and regulatory regions [21], [22]. [23], [24] showed that segmental duplications in human genomes are selectively enriched for genes associated with disease susceptibility, immunity, and defense. Overall, these studies highlighted that annotating segmental duplications in terms of transcription factor binding might aid in understanding functions of genes within these regions. In addition, retrotransposon elements, a major class of transposable elements which duplicate through RNA intermediates that are reverse transcribed and are inserted at new genomic locations, also carry transcription factor binding sites that regulate gene expression [25], [26]. For example, [27] showed with in vivo ChIP experiments that transcription factor p53 binds to human endogenous retrovirus (ERV) long terminal repeats (LTR), that are 100 bp to 5 kb long, with a p53 binding site.

There has been little work in the literature that investigates the effects of multi-reads in ChIP-seq data analysis. [12] provided an example of how discarding multi-reads could result in potential false negatives. [10] briefly discussed that by randomly assigning multi-reads to one of their mapping locations, one can increase the number of detected binding sites. [28] recently studied enrichment of the known repetitive elements as described by the Repbase database [29] and the RepeatMasker [30] scans in ChIP-seq data of histone modifications. Similarly, [31] developed an algorithm for genomic mapping of ambiguous tags which increased read coverage for highly repetitive sequences. However, neither of these thoroughly investigated the effects of multi-reads on overall peak (binding location) detection. Currently, none of the popular ChIP-seq data analysis software [10]–[12], [32]–[42] takes multi-reads into account.

We investigate the effects of discarding multi-reads on two different ChIP-seq datasets: STAT1 binding in interferon-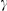-stimulated HeLa S3 cells [10] and GATA1 binding in mouse G1E-ER4 cells [43]. We develop a method for utilizing multi-reads and illustrate that incorporation of multi-reads can lead to an increase of up to 25% in the sequencing depth and identify high quality novel peaks. Location analysis of these peaks reveals that a large fraction of them reside in close proximity to promoters and in genic regions within segmental duplications of the genomes. Furthermore, true peaks are highly enriched for retrotransposon elements such as LINE (long interspersed repetitive elements) and LTR (long terminal repeats). Therefore, they are likely to be critical for constructing comprehensible genetic networks with members in repetitive regions of the genomes. Our computational experiments demonstrate that multi-reads can not only lead to detection of novel peaks in low mappable regions but also improve peak identification in moderate to highly mappable regions. We support our computational experiments by experimental validation of a subset of GATA1 peaks that were only identifiable when multi-reads were incorporated. This leads to identification of novel GATA1 target genes.

## Results

### Multi-reads significantly increase the sequencing depth of ChIP-seq data with 30 to 75mer tags

The two datasets, STAT1 binding in interferon-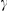-stimulated HeLa S3 cells [10] and GATA1 binding in mouse GATA1-null erythroid cells (G1E-ER4) after genetic complementation with a conditionally active allele of GATA1 (ER-GATA1) [43], and their input DNA controls were downloaded from GEO (http://www.ncbi.nlm.nih.gov/geo/) (accession numbers GSM320736, GSM320737, GSM453997, GSM453998 for STAT1 ChIP and input, and GATA1 ChIP and input samples, respectively). Data from different lanes within an experiment were pooled together. The STAT1 data set has a higher sequencing depth than most published ChIP-seq data sets and is therefore especially suited for studying the effects of multi-reads. Both datasets utilize single end short reads (30 mers for STAT1, 36 mers for GATA1), which is still the current state of the art for ChIP-seq experiments.

We devised a method for allocating multi-reads to a given reference genome. This is motivated by the generative statistical model from [14] that addressed read mapping uncertainty in a principled manner within the context of RNA-Seq. In Table 1 are the total number of reads, percentages of aligning reads, uni-reads, multi-reads, and the rescued-reads (gain in sequencing depth by incorporating multi-reads) for both of the datasets we study. We observe that utilizing multi-reads leads to an increase of 22% (25%) and 17% (19%) in the sequencing depths of STAT1 and GATA1 ChIP (input) samples, respectively. In our mapping procedure, reads mapping to more than 100 locations in the reference genome are discarded due to computational reasons. Despite this, the increase in sequencing depths due to multi-reads is substantial for short read datasets. The last four rows in Table 1 present results for longer reads (unpublished longer read datasets are courtesy of Prof. Qiang Chang at UW Madison). Summaries on MECP2-SET ChIP and input datasets (75 mer single end tags (SETs) from mouse) indicate that multi-reads still constitute a significant issue even with longer reads and they can lead to an increase in sequence depth comparable to the increase in short read datasets. The last two rows are from an experiment with 75 mer paired-end tags (PETs) in mouse. Although there is a significant drop in the percentage of multi-reads, utilizing these reads increases the sequencing depth by 7% for these 75 mer PETs datasets.

**Table 1:** Impact of multi-reads on sequencing depth.

Dataset	# of reads	% Alignable	% Uni-reads	% Multi-reads	% Rescued
STAT1(C)	76,913,219	36.64	29.92	6.72	22.46
STAT1(I)	49,771,625	47.90	38.31	9.59	25.03
GATA1(C)	33,124,216	79.27	67.81	11.46	16.90
GATA1(I)	20,711,007	82.37	69.38	12.99	18.73
MECP2-SET(C)	15,253,906	79.23	65.06	14.16	21.76
MECP2-SET(I)	21,870,009	90.35	78.14	12.21	15.63
MECP2-PET(C)	18,622,331	68.55	64.24	4.31	6.70
MECP2-PET(I)	18,498,899	84.26	78.92	5.34	6.77

In the first column, “(C)” and “(I)” refer to ChIP and input samples, respectively. Percentages in the third to fifth columns are calculated with respect to the total number of reads (the second column). The actual numbers of reads are provided in Table S1. “% Rescued” in the last column is obtained as the number of multi-reads divided by the number of uni-reads and it indicates the gain in sequencing depth due to multi-reads.

### Apparent mappability and GC content sequence biases in multi-read samples

We next evaluated the effect of increase in sequencing depth due to multi-reads in terms of peak detection. We started our exposition by checking for known systematic biases such as mappability and GC content in ChIP-seq data [10], [44]. We divided the genome into small non-overlapping intervals, i.e., bins, of size 50–250 bp as in CisGenome [11] for the downstream analysis of peak detection. We excluded bins which consisted of only the ambiguous base N. Then, for each factor, two bin-level datasets were created using (1) uni-reads only (UR sample) and (2) both uni-reads and multi-reads (MR sample). Further preprocessing involved extending each read to the expected fragment length (200 bp for both datasets) as in [10] and summarizing the total number of reads overlapping each bin. The bin size was selected to match the expected fragment length. Multi-reads contributed fractional counts to multiple locations and these counts were proportional to their estimated alignment probability at each location. The final bin counts were rounded to the nearest integer for modeling purposes since fractional counts were not continuous enough for fitting with a continuous distribution such as the Gamma distribution. We also considered applications of the ceiling and floor functions to fractional counts as alternatives to rounding. These provided upper and lower bounds on the number of reads obtainable from fractional counts, respectively. The overlap of the peak sets under these three strategies were more than 95%. Matching input control samples of the ChIP-seq data were processed similarly to generate matching UR and MR input samples.

**Figure 1:**
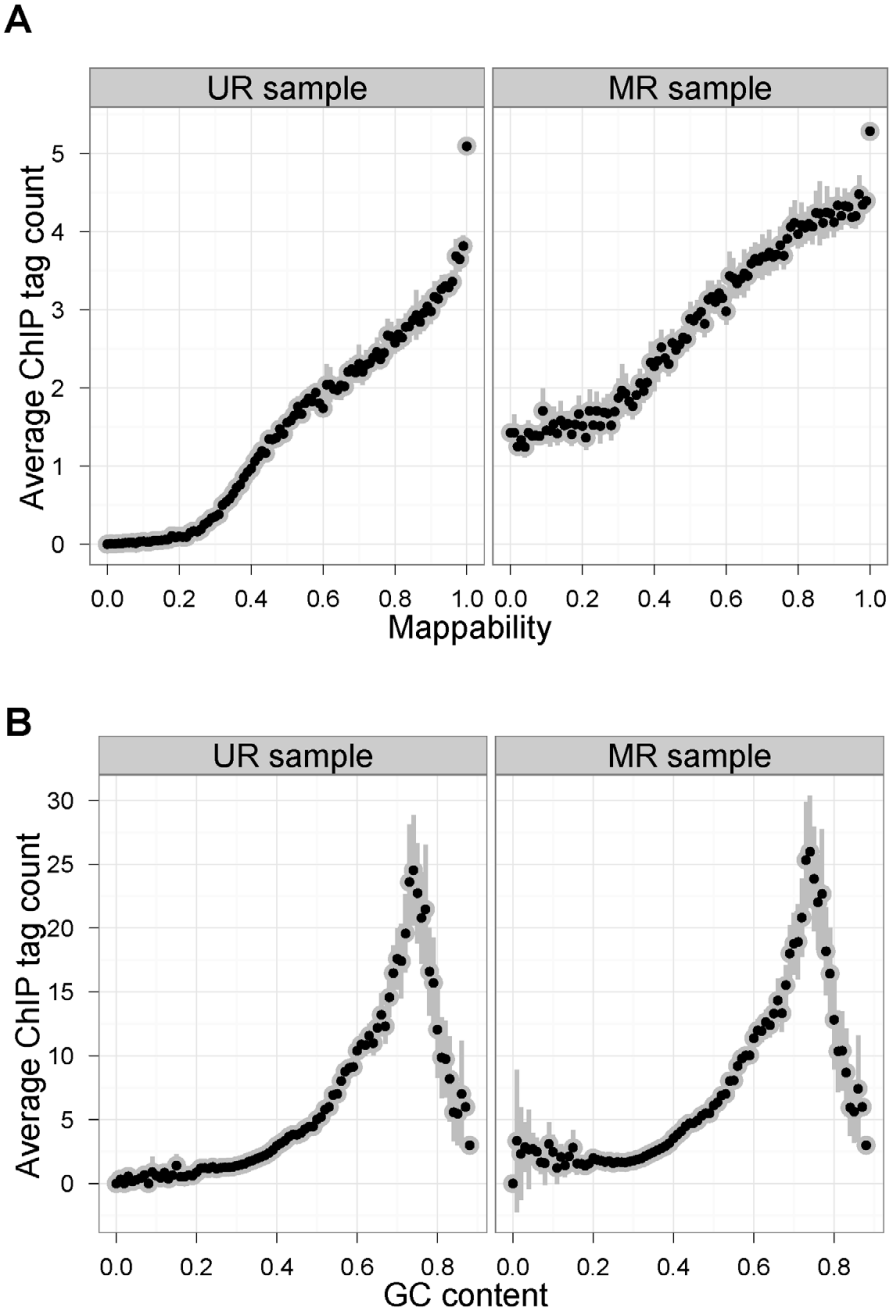
(a) Mappability bias in the STAT1 UR and MR samples. Mean tag counts against mappability (Def 1 - mappability for uni-reads) and GC content in UR and in MR samples, respectively. The patterns observed are typical of ChIP-seq data with 36 mer to 75 mer tags. Figure S2 displays similar patterns for GATA1 UR and MR samples. Mappability plots utilizing Def 2 for the MR samples are provided in Figures S1a and S2a (right panels) and exhibit similar patterns. (b) **GC content bias in the STAT1 UR and MR samples.**

Figures 1A, B display the bin-level average read counts against mappability and GC content for the STAT1 UR and MR samples (Figure S2 displays similar plots for GATA1). Each data point is obtained by averaging the read counts across bins with the same mappability or GC content. It is apparent from Figure 1A that the MR sample still exhibits mappability bias, although to a lesser extent compared to the UR sample in the low mappability range. The GC content biases of both of the samples are comparable. These results indicate that peak detection in the MR samples might also benefit from the use of methods that take into account these apparent sequence biases.

### Multi-read samples reveal significant numbers of additional peaks

We analyzed UR and MR bin-level data for each experiment using our recently developed method MOSAiCS [44] to identify peaks. This method accounts for non-specific sequence biases such as mappability [10] and GC content [45]. It performs comparable to or better than some of the commonly used peak finders such as MACS [32], CisGenome [11], and PeakSeq [10]. Another reason for using MOSAiCS is that currently none of the peak finders readily allow incorporation of multi-reads. The MOSAiCS model fits the UR and MR samples well. Figures S3 and S4 display the goodness of fit (GOF) plots for chromosome 4 of STAT1 and chromosome 9 of GATA1, respectively. Similar fits are observed for other chromosomes. Since MOSAiCS explicitly incorporates mappability as an explanatory variable in a regression framework, we were able to confirm the decrease in the effect of mappability on the model fit of the MR samples by comparing the estimated mappability coefficients in the UR and MR samples. The average coefficient estimates for mappability across chromosomes decreased from 2.94 (UR sample) to 1.95 (MR sample) for STAT1 and 3.72 (UR sample) to 2.75 (MR sample) for GATA1.

**Figure 2:**
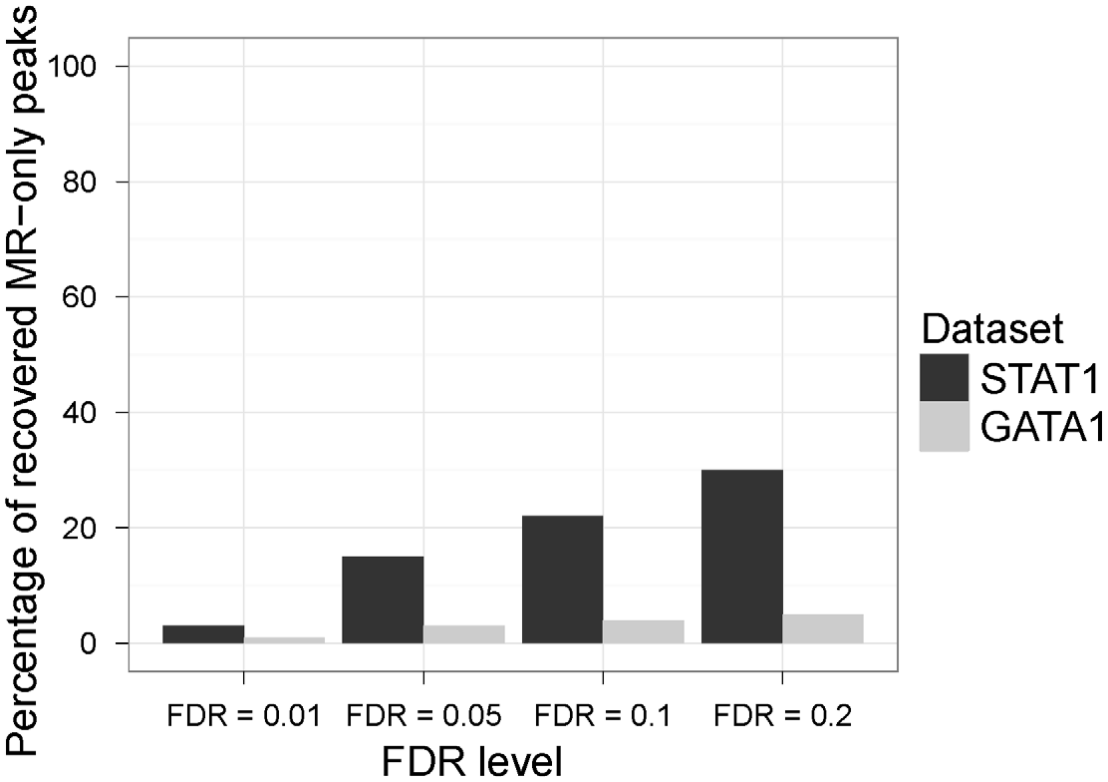
Sensitivity analysis. “Recovered MR-only peaks” refer to MR-only peaks that are defined at FDR level of 0.005 and are detectable by the UR analysis at higher FDR levels.

The final peak lists were obtained by controlling the false discovery rate (FDR) at level 0.05 and filtering out bins with less than 30 ChIP tag counts. Conclusions presented below remain robust to various choices of this tag count threshold. Since the number and the quality of the peaks rely on the FDR level used, we first implemented a sensitivity analysis to evaluate the recovery rate of the UR and MR analysis peaks. We declared peaks at FDR levels of 0.005, 0.01, 0.05, 0.1, and 0.2 and classified the peaks detected at FDR level of 0.005 as UR-only (specific to UR analysis), MR-only (specific to MR analysis), and common peaks. This resulted in 23424 and 3378 MR-only peaks for STAT1 and GATA1, respectively. Then, we evaluated the percentage of the MR-only peaks identified at FDR level of 0.005 and are recovered by the UR analysis at higher FDR levels. We did not calculate the recovery rate of UR-only peaks by the MR analysis since the numbers of UR-only peaks were negligible (2 for STAT1 and 10 for GATA1). Figure 2 displays the results of the sensitivity analysis. As the FDR level increases, the UR analysis can at most recover 30% and 5% of the MR-only peaks for STAT1 and GATA1, respectively.

**Figure 3:**
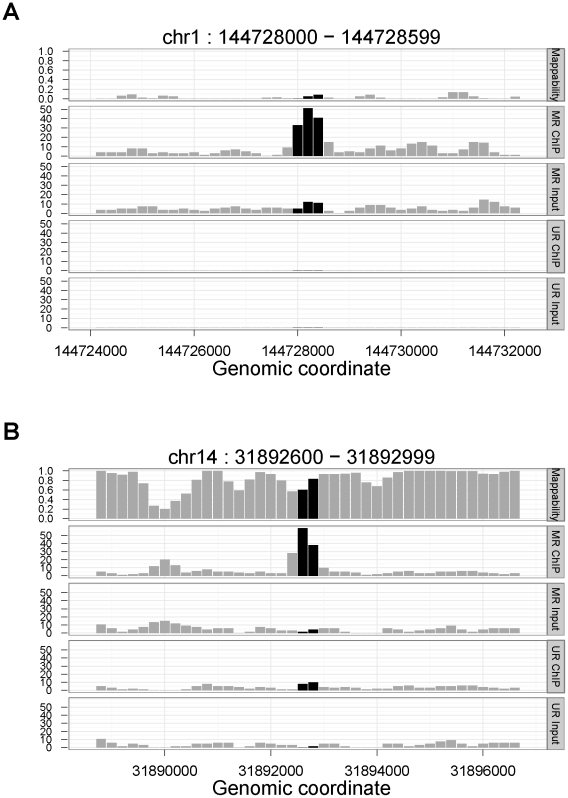
(a) STAT1 MR-only peak in a poorly mappable region. (b) **GATA1 MR-only peak in a moderately mappable region.** Tag count profiles of MR-only peaks with corresponding mappability scores. Peak regions are depicted with black bars.

Figures 3A, B display two examples of MR-only peaks with their MR ChIP, MR input, UR ChIP, UR input, and mappability tracks. The first is a STAT1 peak (Figure 3A) that resides in a poorly mappable region with a peak level mappability of 0.04 and therefore cannot be recovered by the UR analysis that relies only on uni-reads. The second is a GATA1 peak (Figure 3B) and is located in a region with moderate mappability (average peak level mappability of 0.72). It is not identified as a peak in the UR analysis; however since the MR analysis boosts up the tag count of the region by utilizing multi-reads, this peak reaches the required statistical significance level in the MR sample. Of the MR-only STAT1 and GATA1 peaks, 32% and 74% are located in regions with mappability below 0.5, therefore these peaks are not likely to be detected by UR analysis regardless of the sequencing depth.

**Figure 4:**
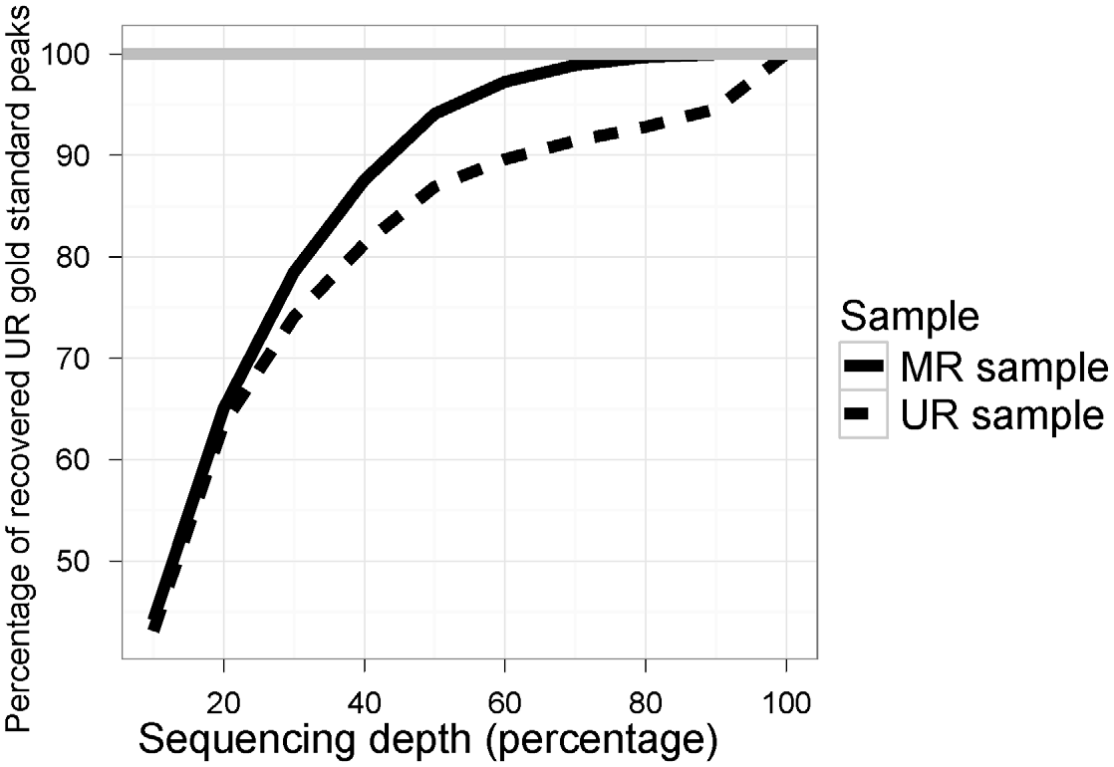
Saturation plot of the STAT1 sample. Percentage of STAT1 UR gold standard peaks recovered by MOSAiCS using sub-sampled UR and MR samples with lower sequencing depths. 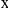-axis refers to the percentage of reads sampled from the full dataset.

To further quantify the advantage of incorporating multi-reads beyond novel peaks that are not identifiable with only uni-reads, we performed the following computational experiment for the STAT1 sample which had higher sequencing depth and was therefore more suitable for this experiment. The idea is similar to the study of saturation in ChIP-seq experiments where the number of identified peaks is plotted as a function of the sequencing depth [10], [37]. We defined peaks identified using all the uni-reads as the UR gold standard peak set. Then, we constructed smaller datasets by sampling from uni-reads and multi-reads and identified peaks using these datasets with lower sequencing depths. Figure 4 plots the percentage of the gold standard UR peaks identified at lower sequencing depths by the UR and MR analysis. We observe that utilizing multi-reads recovers UR gold standard peaks at a faster rate than using only uni-reads. In particular, peak calling using MR sample recovers all the UR gold standard peaks using only 80% of the full dataset. This experiment further solidifies the gains in sequencing depth in Table 1 by illustrating the practical utility of multi-reads in terms of peak finding. When we performed a similar experiment using MR peaks from the full dataset as the gold standard peak set, a significant percentage of MR-only peaks were not detected using only UR sample at any sequencing depth (Figure S5).

For the rest of the comparisons among the MR and UR peak sets, we focused on the highest quality peaks called at FDR level of 0.05 by further filtering UR-only or MR-only peaks. A peak identified only in the MR (UR) analysis is labelled as an MR-(UR-)only peak if its corresponding UR (MR) read count is less than 20 making it highly unlikely for the UR (MR) analysis to identify this peak as a high quality peak. This filtering is further justified by examining MR-only peaks with low and high UR ChIP read counts in more detail. MR-only peaks with low UR ChIP read counts exhibit stronger signal than those with high UR ChIP read counts in the MR sample. Among the STAT1 MR-only peaks, the peaks with low UR ChIP read counts are ranked higher than MR-only peaks with high UR ChIP read counts based on their posterior probability of ChIP enrichment in the MR peak list. Moreover, the average log base 2 bin-level ratio of ChIP over input tag counts of MR-only peaks with low UR ChIP tag counts is 1.72 while those of MR-only peaks with high UR ChIP tag counts is 0.89 (enrichments are computed after scaling ChIP and input to the same total sequencing depth within each sample). Results are similar for the GATA1 MR-only peaks (data not shown).

In addition to the above quality filtering for the UR-only and MR-only peaks, we also screened the MR-only peaks based on their shared multi-reads with other MR peaks. This is to avoid double counting and inclusion of MR-only peaks that are in low mappable segmental duplications. We classified MR-only peaks into three classes: peaks that share high multi-read similarity with another peak (Type-I), peaks that share low multi-read similarity with another peak (Type-II), and peaks that do not share any multi-reads with another peak (Type-III). Type-I MR-only peaks arise if two peaks share a majority of their multi-reads with similar allocation weights and can be potential artifacts of segmental duplications. We observed that 18.8% and 27.6% of the MR-only peaks do not share any multi-reads with another peak (Type-III), for STAT1 and GATA1, respectively. For MR peaks that are also identifiable by uni-reads, these percentages increase to 54.1% and 92.6%. Of the remaining peaks, only 17.3% (STAT1) and 12.3% (GATA1) have a similarity score greater than 0.5 with another peak. We investigated the allocation weights of multi-reads among peaks sharing multi-reads with formal hypothesis testing utilizing both paired t-test and Wilcoxon signed-rank test. This investigation revealed that shared multi-reads might contribute significantly differently to each peak pair despite overall high multi-read similarity. We classified the peaks that are not Type-III as Type-I and Type-II as follows. Peak 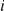 is labeled as Type-II if (a) the sum of its fractional counts, i.e., allocation weights from multi-reads, that do not overlap with peak 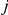 is at least 20; or (b) it has at least 20 uni-reads; or (c) its ratio of sum of fractional counts from unshared versus shared multi-reads is at least 2. Although criterion (a) accounts for 97.2% of the Type-II peaks, we decided to include (b) and (c) based on visual inspection of the multi-read allocation of the peaks. Illustrative examples of scatter plots of allocation weights of MR-only peaks are provided in Figures S6-S7 (Type-II) and Figures S8-S9 (Type-I). As a result, we observed that only 21.8% and 24.4% of the STAT1 and GATA1 MR-only peaks are Type-I peaks. Moreover, 75.9% (STAT1) and 87.4% (GATA1) of the peaks with a similarity larger than 0.5 with another peak are classified as Type-I peaks. In contrast, only 13.5% (STAT1) and 22.6% (GATA1) of the peaks with a similarity smaller than 0.5 are Type-I peaks. Table 2 summarizes the final number of peaks retained in the subsequent downstream analysis after discarding Type-I MR-only peaks. There are no UR-only peaks and multi-reads identify 11% and 36% more high quality peaks for STAT1 and GATA1, respectively. In order to assess the robustness of these results to the peak calling algorithm used, we implemented the conditional binomial (CB) test of CisGenome [11] to handle multi-reads. We processed the CB peaks identified at FDR level of 0.05 with the same procedure applied to MOSAiCS peaks and arrived at the same conclusion: although a large fraction of the peaks are common between UR and MR analysis, MR analysis identifies a significant number of additional peaks. Detailed results of this analysis are in the Table S2. We further compared GATA1 MR-only peaks detected by MOSAiCS with the MACS peaks that were reported in [43] and utilized only uni-reads. Only 127 out of 2146 MOSAiCS MR-only peaks (6%) were in the MACS peak list. In contrast, 97% of MOSAiCS common peaks (5878 out of 6038) were in the MACS list. The 127 MR-only peaks that were detectable by MACS had an average mappability of 0.72 which was significantly higher than the average mappability of 0.31 for the peaks that were not detectable (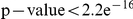). Furthermore, these MACS detectable MR-only peaks had a median ranking of 851 with an interquartile range (IQR) of 609 among the 2146 MR-only peaks, indicating that they are not the strongest signal MR-only peaks. These peaks were also detectable by the MOSAICS analysis of the UR sample at a higher FDR level.

**Table 2:** Summary of UR and MR peaks detected by MOSAiCS.

Dataset	# of UR-only peaks	# of common peaks	# of MR-only peaks
STAT1	0	23175	2546
GATA1	0	6038	2146

### MR-only peaks are from low mappability regions of the genomes but have significant enrichment for known consensus sequences

**Figure 5:**
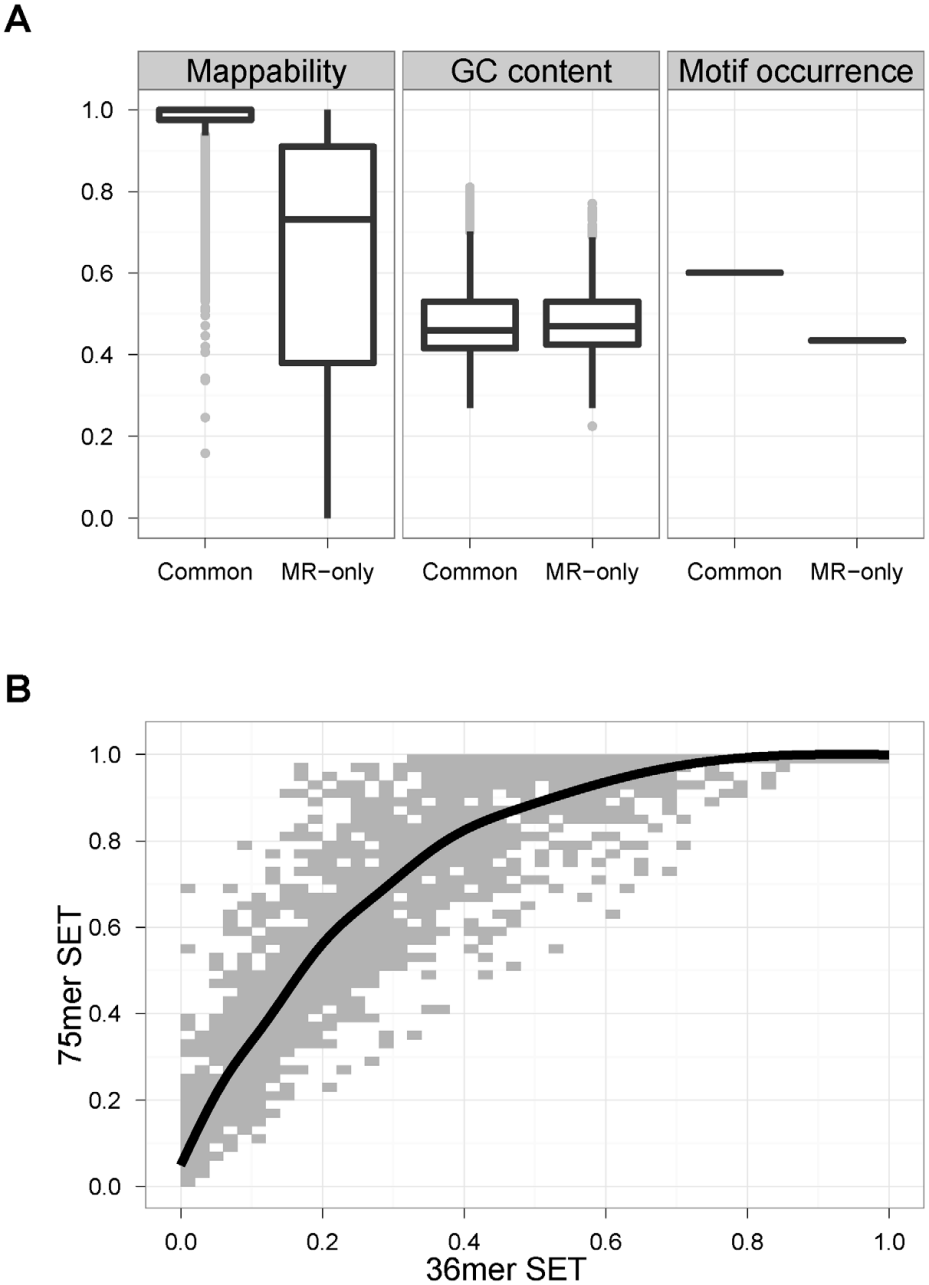
(a) Mappability, GC content, and STAT1 motif occurrence of the common and MR-only peaks. Common refers to common peaks identified by both the MR and the UR samples; MR-only peaks are unique to the MR sample. For the motif occurrence panel, y-axis represents the proportion of peaks with the consensus binding site. (b) **Mappability scores of GATA1 peaks with respect to 75 mer SETs vs. 36 mer SETs.** Scatter plot of mappability of GATA1 MR-only peaks with respect to 75 mer SETs versus 36 mer SETs. The black line is the smooth fit through all the data points. A colored version of the plot where shading in the grids represent frequency of data is provided in the Figure S11.

Figure 5A displays boxplots of mappability (left panel) and GC content (middle panel) of STAT1 common and MR-only peaks (Figure S10 shows the results for GATA1 peaks). The GC content levels are comparable between MR-only and common peaks; however, as expected, MR-only peaks cover a much broader range of mappability and have, on average, lower mappability than common peaks. Next, we investigated how the MR-only peaks would be affected by using longer reads because larger fractions of genomes become uniquely mappable when longer reads are utilized [10]. To assess this, we studied how mappability changes when 75 mer SETs are used instead of 36 mer SETs. Figure 5B displays the scatter plot of mappability scores of GATA1 MR-only peaks (identified using 36 mer SETs) according to 75 mer and 36 mer SETs. Even though mappability improves significantly when longer reads are utilized, indicating that these peaks might eventually become detectable with the uni-read analysis, more than 50% of the GATA1 MR-only peaks still reside in low mappability regions even with 75 mer SETs. The median mappability of GATA1 MR-only peaks increase from 0.27 (IQR = .40) to 0.67 (IQR = .59) when 75 mer SETs are used instead of the 36 mer SETs.

Next, we compared enrichment ratios of the peaks by taking an average of bin-level ChIP to input ratios in the log base 2 scale. We observe that, overall, common peaks tend to have higher enrichment ratios compared to MR-only peaks; average enrichments, i.e., fold-changes in the log base 2 scale, are 2.05 (2.89) and 1.72 (2.03) for STAT1 (GATA1) common and MR-only peaks, respectively. However, further analysis reveals that 77% and 38% of the GATA1 MR-only peaks have enrichment ratios larger than 1 and 2, respectively. For STAT1, 68% and 30% of the MR-only peaks fall in these categories.

We also compared the peak sets in terms of their enrichments for the known binding consensus sequence of the corresponding factors. We scanned the STAT1 peaks with FIMO [46] using the two known STAT1 position weight matrices from JASPAR [47]. For GATA1, we counted the occurrence of the consensus motif [A/T]GATA[A/G] [48] in the peak regions. Right panel of Figure 5A displays the STAT1 motif occurrences of common and MR-only peaks. Figure S10 (right panel) exhibits similar results for GATA1. Motif enrichments in the MR-only peak sets are lower compared to the enrichment observed for the common peaks. This is potentially due to uncertainty in the mapping of reads that contribute to these peaks. However, the observed motif enrichments in both the STAT1 and the GATA1 MR-only peak sets are much higher than one would expect by chance (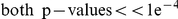).

### Genome-wide annotation reveals enrichment of MR-only peaks in segmental duplications

**Figure 6:**
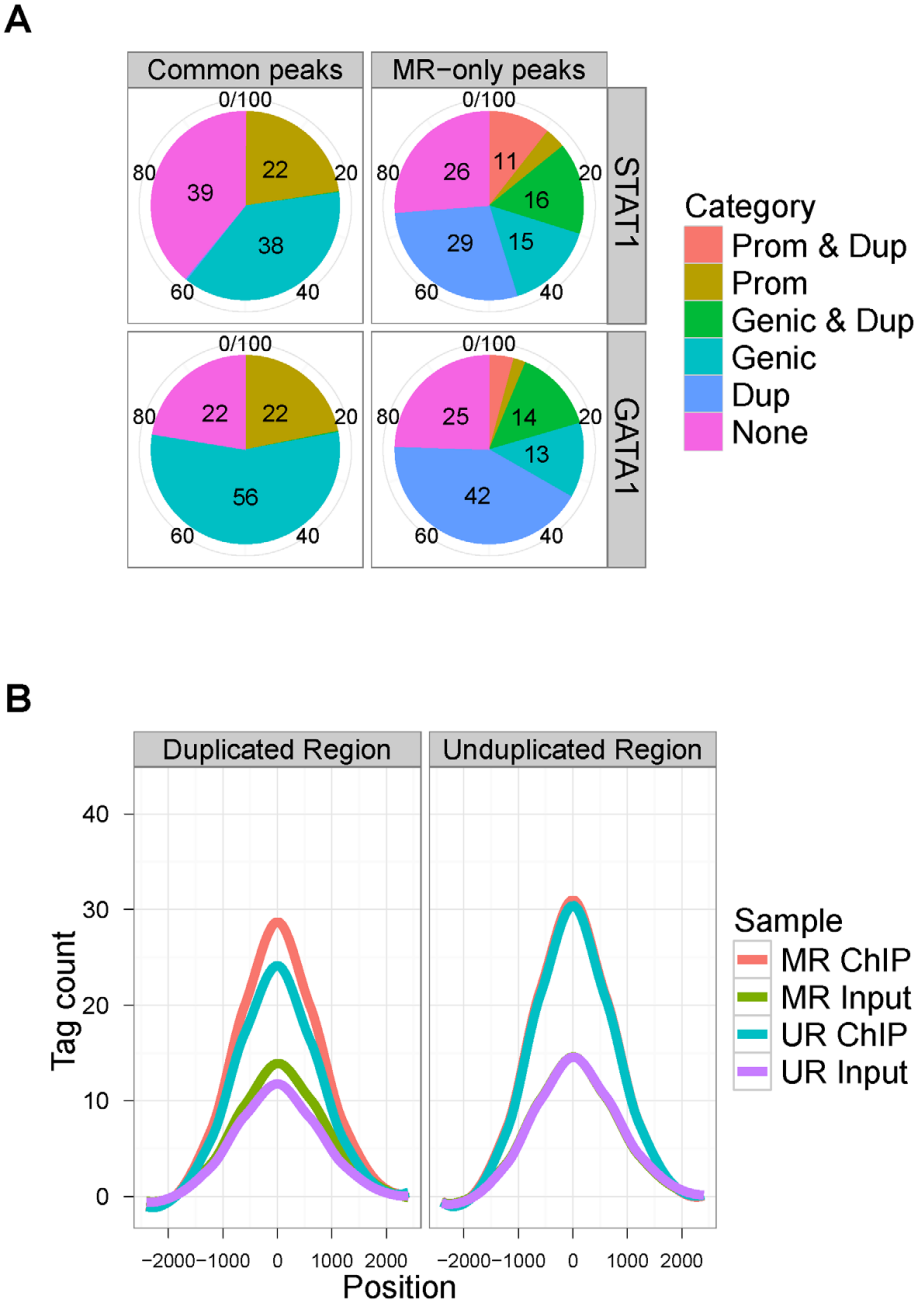
(a) Annotation of common and MR-only peaks with respect to TSS and duplicated regions. Categories are: Prom & Dup: peaks that are in promoter regions (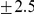 kb of TSS) of RefSeq genes that reside in segmental duplications; Prom: Peaks in promoter regions (excludes peaks in Prom & Dup); Genic & Dup: peaks that are within [−10 kb of TSS, +1 kb of TES] of RefSeq genes that are in segmental duplications (excludes peaks in Prom & Dup); Genic: peaks that are within [−10 kb of TSS, +1 kb of TES] of RefSeq genes (excludes peaks in Genic & Dup, Prom, and Prom & Dup); Dup: peaks that are in segmental duplications (excludes Prom & Dup and Genic & Dup); None: peaks that do not fall into any of the other defined categories. Numbers within the pie charts indicate the percentages of peaks in each category. (b) **Aggregation plots depicting STAT1 uni-read and multi-read ChIP and input signals across** 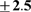 **kb of TSS of expressed genes in duplicated and unduplicated regions** Tag counts within each profile are normalized by subtracting average counts of two bins at both ends of the boundary. ChIP tag counts in 1 kb region around the TSSs within segmental duplications (left panel) increase by an average of 24.69 tags in the MR ChIP sample compared to the UR ChIP sample. The corresponding increase in the input tag counts is only 9.93 tags. In contrast, for the TSSs that reside outside of the segmental duplications (right panel), ChIP and input tags counts increase on average by 3.08 and 0.39 tags in the MR sample compared to UR sample.

As we discussed in the Introduction, there is a growing literature that highlights the biological importance of segmental duplications [23], [24], [49]. One of the findings is that segmental duplications are enriched for genes involved in immunity and, therefore, could be potential targets for transcription factor binding. We next assessed to what extent common and MR-only peaks appear in segmental duplications of the genomes. For this analysis, we utilized segmental duplication data from the UCSC Genome Browser database [50] and carried out a location analysis on the peak lists. Pie charts in Figure 6A display location analysis results for STAT1 and GATA1, respectively. MR analysis identifies peaks in all categories. The percentages of MR-only peaks that are in the “None” category are not drastically different than that of the common peaks. A large percentage of MR-only peaks reside in segmental duplication regions (54.91% for STAT1 and 60.58% for GATA1) with a substantial amount located in promoter (10.60% and 4.19% for STAT1 and GATA1, respectively) and genic regions of genes (15.71% and 14.17% for STAT1 and GATA1) within these segmental duplications. Next, we annotated the peaks in the "None" category in terms of interspersed repeats and low complexity DNA sequences in the human and mouse genomes utilizing RepeatMasker [30]. For STAT1, 67% of the 8782 common peaks and 95% of the 667 MR-only peaks map to at least one of these types of repeats. In particular, MR-only peaks are enriched in the long terminal repeats (LTR) category compared to common peaks. Percentages of peaks in the LTR category are 22.6% and 58.5% for the common and MR-only peaks, respectively. For GATA1, 54% of the 1347 common peaks and 76% of the 526 MR-only peaks map to at least one of these types of repeats. In addition, MR-only peaks are enriched in the long interspersed repetitive elements (LINE) (9.3% of the common peaks, 22.6% of the MR-only peaks) and LTR (16.5% of the common peaks, 45.6% of the MR-only peaks) categories compared to common peaks. In contrast, common peaks are enriched in simple repeat and short interspersed repetitive elements (SINE) category. Percentages of common peaks among the "None" category that are in simple repeat and SINE categories are 12.5% and 24.3% compared to 7.2% and 7.4% for the MR-only peaks in these categories. Further results on the annotation of the common and MR-only peaks in terms of repeat elements other than segmental duplications for STAT1 and GATA1 are available in Tables S7 and S8, respectively.

To further explore STAT1 peaks in UR and MR samples in terms of segmental duplications, we compared the average tag count profiles at promoters of expressed genes [51] in duplicated and unduplicated regions in Figure 6B. Of the 10913 expressed genes, 1862 (17%) are located within segmental duplications. The profiles for the matching input DNA controls were also included for relative enrichment comparison between STAT1 ChIP and input samples. These profiles are normalized using the average of four bins at the start and end of the profiles. Both the UR and MR samples have comparable tag counts at promoters of expressed genes in unduplicated regions. In contrast, STAT1 ChIP MR sample exhibits increased signal at promoters in duplicated regions relative to input MR sample. Specifically, 1 kb regions around the transcription start site (TSS) of the expressed genes in duplicated regions gain on average 24.69 and 9.93 tags in MR ChIP and input samples, respectively, compared to UR ChIP and input samples. In contrast, the gains in unduplicated regions are only 3.08 and 0.39 tags compared to UR ChIP and input samples.

We used the DAVID tools of [52], [53] to further annotate the MR-only peaks. For STAT1, we applied DAVID to the group of 102 expressed genes with at least one MR-only peak and no common peaks in their promoters. This analysis revealed significant enrichment of these genes for response to DNA damage, transcription activity, regulation of gene expression, apoptosis, programmed cell death, and intercellular signaling cascade. For GATA1, the set of expressed genes with an MR-only peak was too small. Instead, we applied DAVID to the set of genes with at least one MR-only peak and no common peaks within 10 kb upstream of TSS and 2 kb downstream of transcription end site (TES) excluding exons. DAVID analysis of such 340 genes identified significant enrichment for immune/defense response, immune system development, regulation of apoptosis, hemopoiesis, and SAND domain. These results agreed well with the observation that the segmental duplications are selectively enriched for genes associated with immunity and defense [23], [24].

### Experimental validation of MR-only peaks confirms novel GATA1 target genes

**Figure 7:**
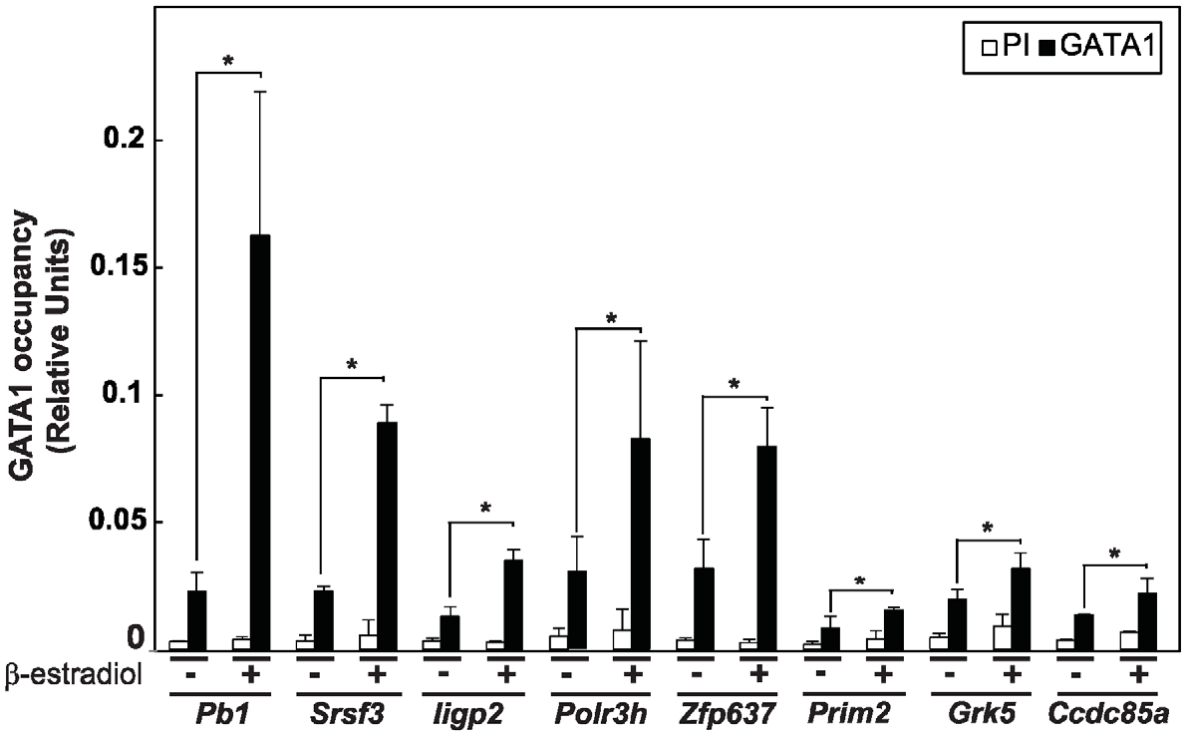
Experimental validation of GATA1 MR-only peaks. Quantitative real-time ChIP analysis of a subset of MR-only peaks in 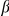-estradiol untreated (−) and 24 hrs treated (+) G1-ER-GATA1 cells based on three independent biological replicates (*: 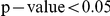). The Preimmune (PI) values did not exceed 0.006.

We selected 13 GATA1 MR-only peaks for experimental validation for GATA1 occupancy with quantitative ChIP assays and real-time PCR. Peaks selected for validation contained a [A/T]GATA[A/G] motif, resided within promoter or genic regions of a RefSeq gene, and had a mappability value between 0.5 and 1. Eighteen percent of the GATA1 MR-only peaks satisfied the two former requirements. The mappability constraint was necessary for designing unique primers for the real-time PCR analysis of the peaks. The quantitative ChIP experiments were performed in the G1E-ER-GATA1 cells, which are pro-erythroblast cells derived from mouse ES cells in which the Gata1 gene was disrupted via homologous recombination and further engineered to express a conditionally active estrogen receptor (ER) ligand binding domain fusion to GATA1 (ER-GATA1). When 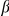-estradiol is added to the culture medium (+EST), the ER-GATA1 fusion protein gets activated and binds to GATA1 specific sites. Figure 7 displays quantitative ChIP analysis of GATA1 occupancy of the eight validated peaks, where +EST and -EST refer to relative occupancy with respect to input DNA with or without 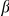-estradiol treatment (Figures S12, S13, S14, S15, S16, S17, 18, and S19 display raw data tracks for these peaks). We observe a significant increase in GATA1 occupancy in +EST compared to -EST for these MR-only GATA1 targets. Of these 13 peaks, 9 are located in the lower 50th percentile of the full peak set obtained with the MR sample in terms of their peak scores (median rank of the 13 peaks is 4661 out of 8184 peaks from the MR sample). A scatter plot of average log base 2 ChIP to Input ratio versus ChIP count for all the GATA1 MR-only peaks are provided in Figure S20. Although this validation rate of 61.5% is not representative of all MR peaks since the selected peaks are from promoter/genic regions and are required to have a GATA motif, it agrees well with rates one might observe in the lower half of a ranked ChIP-seq peak list. Previous studies exhibited that the validation rate may drop to 61.5% even at the bottom of the upper 50th percentile of a UR sample peak list [5]. In addition to our validation experiments of MR-only peaks, we also performed validation experiments for 7 [A/T]GATA[A/G] sites that resided in low mappable regions and were not predicted to be MR peaks. None of these 7 regions exhibited an increase in GATA1 occupancy in +EST compared to -EST (Figure S21), confirming that they are true negatives.

We next analyzed the expression of the genes corresponding to the above validated peaks in 24 hr 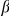-estradiol treated G1E-ER-GATA1 cells using microarray data generated from 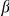-estradiol treated and control cells as described in [5]. Upon 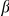-estradiol-treatment and GATA1 activation, these genes exhibited a fold change of 0.9 to 4.9 in expression. This confirms that GATA1 binds to these MR-only peaks and triggers expression of their corresponding genes during GATA1 mediated maturation of pro-erythroblasts. Even though these genes are not direct erythroid maturation factors, the megakaryocyte-erythrocyte progenitors express these factors at substantial levels as evidenced in the BioGPS analysis [54]. These validated genes are chromatin modifiers, m-RNA splicing factors, zinc finger proteins and are mainly involved in transcriptional regulation and signal transduction. They may further contribute to the expression of erythroid specific genes/factors after being activated in the early phase of erythroid maturation.

## Discussion

We investigated the shortcomings of discarding multi-reads in ChIP-seq analysis and illustrated how incorporating multi-reads can improve detection of binding sites in highly repetitive regions of genomes. Multi-reads lead to identification of novel binding sites that are located in highly repetitive and low mappability regions and are not identifiable with uni-reads alone. They further contribute to effective utilization of uni-reads so that more peaks can be detected with lower sequencing depths. Utilizing location analysis and biological annotation, we further showed that a substantial fraction of peaks specific to multi-read analysis are located in segmental duplications of the human and mouse genomes, and attributed to genes that are well associated with immunity and defense.

Since multi-reads arise automatically in ChIP-seq experiments, our pipeline does not require any additional experiments for utilizing these reads. Once multi-reads are appropriately converted into counts by an application of the multi-read allocation algorithm, peak calling might be performed with any method that can handle bin or nucleotide level count data; however, since many of existing software start the analysis with aligned or raw tag files, these would need to be modified. To accommodate some of the existing software that rely on aligned read files (or alignment results in the bed format), we developed a script that rounds the multi-read weights to the nearest integer and adds the ones that round up to 1 to the original alignment files as pseudo reads so that they can be utilized. This procedure is equivalent to (1) allocating each multi-read to the location that it maps to with the largest weight; (2) filtering out multi-reads with 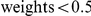 since they round to 0; and (3) ignoring weight information (degree of confidence for multi-reads allocation). Although this implementation decreases the number of utilized multi-reads by about a half (for GATA1), it still leads to a significant increase in the sequencing depth compared to using uni-reads alone. An application of this strategy with the MACS algorithm [32] was able to identify 37% of the MR-only peaks identified by the MOSAiCS MR analysis. This set included the 3 true positive and 2 false positive peaks that we validated with the quantitative real time ChIP analysis.

We showed that the overall conclusions of utilizing multi-reads agree well when peak calling is performed either with MOSAiCS [44] or CisGenome's conditional binomial model [11]. Almost all of the the published ChIP-seq studies in GEO (http://www.ncbi.nlm.nih.gov/geo/) utilize short reads (25–36 mers) and we have observed that multi-reads can lead to a substantial increase in the sequencing depths of such datasets. A thorough investigation of peaks that were detectable only with multi-reads highlighted that a significant fraction of these peaks still have low mappability even when 75 mer SETs are used. Therefore, utilization of multi-reads is also likely to improve the analysis of data from longer and/or paired-end reads. The two factors we studied are not particularly known to bind to repetitive regions. However, there are many examples of DNA binding proteins, e.g., MECP2, KAP1, that selectively bind to repetitive regions. Our pipeline should even have a higher impact in the analysis of such datasets. We have initially implemented our methodology for use with ChIP-seq data from the Illumina Genome Analyzer platform; however it is straightforward to adapt it for use with other high-throughput sequencing platforms.

A related question is whether utilizing a more flexible definition of uni-reads by relaxing the alignment criterion can provide a similar utility to that of multi-reads in terms of sequencing depth. Our computational experiments (data not shown) indicate that more flexible definitions of uni-reads (e.g. single best alignment of each read with at most 3 mismatches) can increase the sequencing depth and lead to identification of more peaks. However, such applications fail to identify high signal MR-only peaks that a multi-read analysis can identify. For example, defining uni-reads by considering the single best alignment of each read with at most 3 mismatches increases the sequencing depth by 12% for GATA1 but can only identify 2.6% of the GATA1 MR-only peaks.

Significant fractions of eukaryotic genomes are composed of repetitive regions, e.g., more than half of the human genome. Therefore, functional properties of the repetitive regions of genomes are of significant biological interest. In particular, genomic repeats play important roles in functioning and evolution of transcriptional regulatory networks [55], [56]. [56] illustrated that binding sites embedded in genomic repeats are associated with significant regulatory expansions throughout the mammalian phylogeny. Therefore, analysis and/or re-analysis of available or future ChIP-seq datasets with our multi-read approach is expected to reveal fundamental insights into functional properties of highly repetitive regions of the genomes.

## Methods

### Allocating multi-reads

The computation of weighted alignments for the reads in each data set was performed in two steps. First, a short-read alignment tool was used to establish a set of candidate alignments for each read against the reference genome. Second, an iterative alignment reweighting algorithm was used to establish probabilities for each candidate alignment. For the first step, we used the Bowtie aligner [57] to align reads against the appropriate reference genome (human HG18 or mouse MM9). The parameters for Bowtie were set such that for each read, all alignments with at most 2 mismatches were reported. Reads with 100 or more such alignments were filtered out. For all samples except STAT1, reads were obtained from the FASTQ-formatted files produced by the Illumina pipeline. For STAT1, reads were extracted from the output of the ELAND aligner (reads with ELAND tags “QC” or “RM” were filtered out).

Given a set of candidate alignments for each read, we used a novel alignment weighting algorithm to assign probabilities of being correct to each alignment. The strategy we used can be thought of as an iterated version of the “rescue” technique described in [13], or a heuristic version of the Expectation-Maximization method used for estimating expression levels from RNA-Seq data [14]. During the algorithm, we maintain a list of (possibly non-integer) counts of the number of reads assigned to start at each position in the genome. Strand information for each alignment was ignored such that the left-most coordinate of each alignment was defined as its starting position. On each iteration of the algorithm, we reallocated the fraction of a read assigned to each of its possible starting positions using the counts from the previous iteration. The fraction of a read assigned to a given position 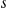 was defined to be proportional to the number of reads assigned within a length 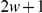 interval centered on 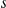 (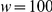 for this study). More precisely, for a read with 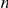 possible starting positions, 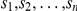, the fraction of the read assigned to position 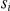 was computed as 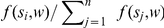, where
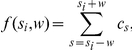
and 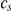 is the count of reads assigned to position 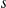. The algorithm was initialized by setting 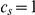, for all positions 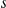 and was repeated for a fixed number of iterations (200 in this study). The fraction of a read allocated for each alignment in the final iteration was used as the probability of that alignment being correct. In this allocation procedure, the choice of 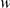 controls the degree at which multi-read allocation is affected by uni-reads. Therefore, setting 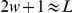, where 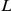 is the fragment size (200 bp for both STAT1 and GATA1 datasets), ensures that uni-reads and multi-reads within a given bin correspond to the same binding event. We considered setting 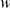 to 25, 50, and 100 bp, respectively, and observed that, although there is high overlap among the peak sets obtained with different 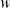 (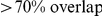), 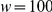 captures the largest number of true positives and smallest number of false positives in our validation set.

For efficient storage and retrieval of the number of reads mapped to each position in the genome, a binary tree structure, similar to that of a Fenwick tree [58] was used. This structure allows for 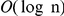 time updates of read counts and 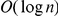 time computations of the cumulative sum of counts within any interval, where 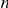 is the total number of genomic positions for which counts are recorded. For space efficiency, only genomic positions at which at least one read alignment started are stored in the tree. The multi-read allocation algorithm is available at http://www.stat.wisc.edu/ 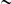
keles/Software/multi-reads/ in the form of a C++ program.

### Mappability for 36mer and 75mer SETs

Consider a tag length of 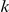 and a fragment length of 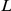. Let 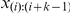 denote the 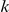
mer starting at position 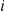 and ending at position 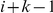 from 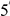 to 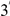. Let 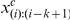 denote the 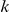
mer starting at position 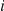 and ending in 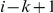 in the other strand. Nucleotide level mappability is defined as in [10]:
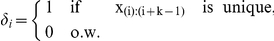



We can similarly define mappability for a position in the reverse strand as:
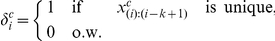



Note that 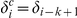. In the pre-processing of ChIP-seq data, as a result of fragment extension, the total number of observed counts at position 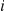 could be contributed by forward strand tags that originate between positions 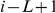 and 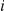 or reverse strand tags that originate between positions 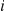 and 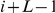. Therefore, we modify the definition of mappability at position 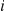 as follows for single-end tags:
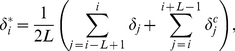
(1)

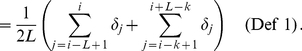
(2)


Mappability plots in Figure 1A, Figure S1a, and Figure S2a (middle panels) are based on the above definition of mappability to highlight the effect of multi-reads on low mappable regions and allow direct comparison with uni-reads.

When we are utilizing multi-reads in the actual statistical analysis, the definition of nucleotide level mappability is modified to take into account the fact that 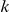
mers mapping to less than 100 locations can generate non-zero counts. This can be achieved by defining 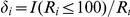 where 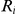 is the number of times 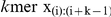 occurs in the genome. We will refer to bin-level mappability utilizing this 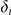 as Def 2. Figures S1a and S2a (right panels) illustrate that the mappability bias is also apparent for the MR samples with this modification on mappability to take multi-reads into account.

Finally, the mappability score 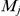 for bin 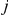 is the average of mappabilities (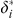) of positions that are within this bin. The GC content at the bin level is calculated similarly by changing 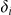 to be GC content of the 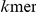 starting at position 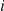.

### MOSAiCS

MOSAiCS is based on a two-component mixture model where data from unbound bins, i.e., background bins, are characterized with a negative binomial regression model that accounts for mappability, GC content, and input counts. Data from bound bins are modeled with a mixture of two negative binomials. Let 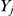 denote ChIP tag counts, 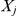 input tag counts, 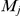 and 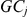 mappability and GC content, respectively. Define 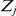 to be an unobserved random variable representing whether the bin is bound or not. Then, MOSAiCS assumes that
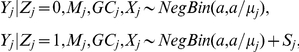
where 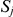 is a mixture of two negative binomial distributions, i.e., 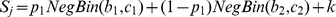, where 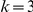 is a constant that represents the minimum tag count observable in a bound region; 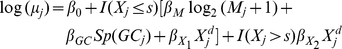 and 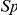 is a piecewise linear B-spline model with knots at the first and third quartiles of GC content. We had previously shown that this piecewise linear B-spline model characterizes the dependence of background tag counts on GC content well [44]. 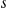 and 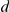 are tuning parameters. For all the datasets we have used MOSAiCS on, 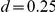 works best. Optimal 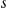 was chosen among 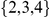 for each chromosome based on BIC scores. The R package mosaics implements this model (available from Bioconductor [59]) and provides parameter estimates and posterior probabilities depicting the probability that a given bin is bound. False discovery rate (FDR) is then controlled at the desired level utilizing these posterior probabilities. Contiguous bins declared as bound are merged as peaks.

### Classification of MR-only peaks based on their shared multi-reads

We classified the MR-only peaks into three classes, Type-I, II, and III, based on their shared multi-reads with the following procedure in a high throughput fashion.

1. For each peak, we identified the set of reads that map to its peak region. As expected, no uni-reads were shared between any two peaks. There were cases where the same multi-read was mapped to multiple locations within a peak (albeit with different weights). We collapsed reads mapping to a peak to a unique set of reads and combined weights from multiple mappings of the same read within the peak region.

2. For every pair of peaks, we counted the number of shared multi-reads and computed a similarity score between them based on their multi-reads. Although two peaks can share a substantial number of multi-reads, the weights of the reads may be substantially different for the two peaks. For example, if a multi-read is shared by peaks 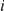 and 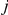 but its weight for peak 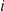 is 0.9 and is 0.1 for peak 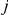, then the two peaks can be more different, compared to the case when the weights are 0.6 and 0.4 for the two peaks, respectively. Therefore, we weighted the contribution of each multi-read to the similarity score by the difference in its weights in the two peaks. Then, the similarity between peaks 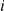 and 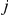 is defined as 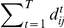 where 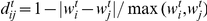 and 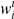 is the fractional count of read 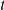 in peak 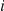. For the above example, this results in 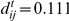 if 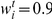 and 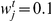 and 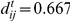 if 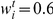 and 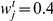.

Under this similarity definition, the self-similarity (diagonal elements of the similarity matrix), is just the total number of reads in the peak. In addition, if the weights for each overlapping multi-read between peaks 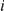 and 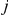 are all 0.5, then the similarity is again the total number of overlapping reads. We normalized the similarity scores between peaks 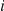 and 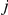 by the maximum of the number of reads in peaks 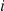 and 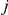 to make the similarity scores comparable across peaks.

3. We further assessed the degree of similarity of peak pairs sharing multi-reads with a paired t-test of the multi-read weights. Specifically, we tested whether the average weights of the shared multi-reads are the same for each peak of the pair. This analysis suggested that even for peaks with high similarity based on the above definition (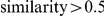), the weights could contribute significantly differently to the two peaks. We obtained similar results when the paired t-test is replaced with the non-parametric Wilcoxon signed-rank test.

4. Finally, we classified a peak as Type-II if (a) the sum of its fractional counts that do not overlap with peak 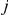 is at least 20; or (b) it it has at least 20 uni-reads; or (c) if its ratio of sum of fractional counts from unshared versus shared multi-reads is at least 2.

Detailed results of the similarity analysis and formal hypothesis testing of shared multi-read allocation weights are provided in Tables S3, S4, S5 and S6.

### Conditional binomial test

For each bin 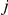, let 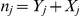 denote total tag counts. The conditional binomial test of CisGenome [11] builds on the fact that under the independence of ChIP counts 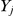 and input counts 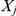, the conditional distribution of 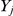 given 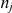 follows a binomial distribution with parameters 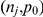. 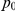 is estimated by 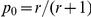, where 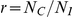, 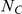 and 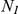 are the total ChIP and input tag counts across the bins with total counts smaller than or equal to 1. Under this null model of no binding, a p-value is computed for each bin and overall FDR can be controlled at any desired level. Contiguous bins declared as bound are merged as peaks.

### Saturation analysis

STAT1 UR gold standard peaks are defined as the peaks detected by MOSAiCS at FDR level of 0.005 based on the whole STAT1 UR sample. Then, we sampled 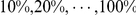 of uni-reads and multi-reads, respectively, and set the UR and MR samples as the sampled uni-reads and combined uni-reads and multi-reads, respectively. We also made sure that the samples obtained at higher sampling percentages are super sets of the samples with lower sampling percentages. After the construction of UR and MR samples of increasing sequencing depth, we detected peaks in these samples with MOSAiCS using the same parameter setting as in the construction of the UR gold standard peak set. We then compared the overlap of the identified peaks sets with the UR gold standard peak set.

### Segmental duplications

Segmental duplications are defined as 1 kb or longer repeats with at least 90% similarity to another region within the genome [24], [60]. We downloaded segmental duplication data for human HG18 and mouse MM8 from the UCSC Genome Browser database (‘genomicSuperDups’ table in the ‘Segmental Dups’ track) [50]. Then, duplicated segments of MM8 were lifted over to MM9 using the lift-over tool of the UCSC Genome Browser database.

### Motif analysis

We scanned the peak sets with both versions of STAT1 motifs (ID: MA0137.1 and MA0137.2) using the position weight matrices in the JASPAR database [47]. Scoring on each peak set was conducted with the FIMO tool of the MEME suite [46], [61]. FIMO evaluates the significance of each subsequence in a given dataset by comparing the likelihoods of the subsequence under the position weight matrix model and a background model. For each peak set, we allowed the background model to be estimated from the sequences of all the peaks. The resulting p-values were adjusted by controlling peak level FDR at 0.1 to take into account the differences in peak widths, where median widths of common and MR-only peaks were 600 and 400 bp, respectively. For GATA1, we counted the occurrence of the GATA1 consensus sequence [A/T]GATA[A/G] [48] in the peak regions. The median widths of both the common and MR-only peaks were 400 bp.

We assessed the significance of motif occurrences by estimating a null distribution of motif occurrence for each factor, separately. We repeated the following null peak set generation 10,000 times for each of the MR-only peak lists. First, for each peak in the peak list, we randomly sampled a region matching the actual peak in terms of width, mappability, and GC content from the same chromosome. After sampling as many peaks as the number of peaks in the actual dataset, we scanned the peaks for motif occurrence with the procedure used for the actual dataset. We then reported the proportion of peaks with the motif in each of the 10,000 simulated datasets. A p-value for each MR-only peak list was obtained by taking the proportion of number of simulated peak sets out of 10,000 with a motif occurrence proportion greater than that of the actual MR-only peak list's proportion.

### Quantitative ChIP assay

Quantitative ChIP analysis was conducted in G1-ER-GATA1 cells with (+EST) and without (-EST) 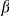-estradiol treatment and validated as described in [62]. The PCR primers are provided in Table S9. P-values for assessing the change in GATA1 occupancy upon 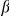-estradiol treatment are based on one-sided t-tests.

## Supporting Information

Figure S1 **Mappability and GC content sequence biases in the STAT1 UR and MR samples.** Mean tag counts against mappability and GC content in the UR and MR samples, respectively. The patterns observed are typical of ChIP-Seq data with 36 mer to 75 mer tags. “Def 1” and “Def 2” indicate the definitions of mappability for the UR and MR samples, respectively.
(PDF)

Figure S2 **Mappability and GC content sequences biases in the GATA1 UR and MR samples.** Mean tag counts against mappability and GC content in the UR and MR samples, respectively. The patterns observed are typical of ChIP-Seq data with 36 mer to 75 mer tags. “Def 1” and “Def 2” indicate the definitions of mappability for the UR and MR samples, respectively.
(PDF)

Figure S3 **MOSAiCS goodness of fit for the STAT1 UR and MR samples.** **(a)** Goodness of fit for the UR sample. **(b)** Goodness of fit for the MR sample. Both axes are in log10 scale.
(PDF)

Figure S4 **MOSAiCS goodness of fit for the GATA1 UR and MR samples.** **(a)** Goodness of fit for the UR sample. **(b)** Goodness of fit for the MR sample. Both axes are in log10 scale.
(PDF)

Figure S5
**Saturation plot of the STAT1 sample.** Percentage of STAT1 MR gold standard peaks recovered by MOSAiCS using sub-sampled UR and MR samples with lower sequencing depths. 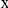-axis refers to the percentage of reads sampled from the full dataset.(PDF)

Figure S6
**Scatter plot of multi-read weights for a Type-II peak # 1.** Peak 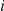 is a Type-II MR-only peak (chr4: 145,711,000 - 145,711,199) with a total of 54 mapping reads, 4 of which are uni-reads (circled in blue). It shares a maximum of 9 reads (circled in green) with peak 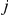 with a maximum multi-read similarity of 0.0144. The sums of its fractional counts from unshared and shared multi-reads with peak 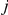 are 28.25 and 0.47, respectively. Uni-reads of peak 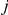 are circled in magenta.(PDF)

Figure S7
**Scatter plot of multi-read weights for a Type-II peak # 2.** Peak 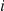 is a Type-II MR-only peak (chr3: 96,290,200 - 96,290,799) with a total of 328 mapping reads, none of which are uni-reads. It shares a maximum of 236 reads with peak 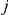 with a maximum multi-read similarity of 0.516. The sums of its fractional counts from unshared and shared multi-reads with peak 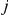 are 73.94 and 17.30, respectively. Uni-reads of peak 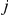 are circled in magenta.(PDF)

Figure S8
**Scatter plot of multi-read weights for a Type-I peak # 1.** Peak 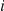 is a Type-I MR-only peak (chr7: 9,183,800 - 9,184,199) with a total of 200 mapping reads, none of which are uni-reads. It shares a maximum of 161 reads with peak 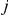 with a maximum multi-read similarity of 0.717. The sums of its fractional counts from unshared and shared multi-reads with peak 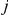 are 8.97 and 40.45, respectively.(PDF)

Figure S9
**Scatter plot of multi-read weights for a Type-I peak # 2.** Peak 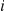 is a Type-I MR-only peak (chr12: 19,040,000 - 19,040,399) with a total of 343 mapping reads, none of which are uni-reads. It shares a maximum of 312 reads with peak 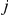 with a maximum multi-read similarity of 0.762. The sums of its fractional counts from unshared and shared multi-reads with peak 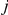 are 7.73 and 53.68, respectively.(PDF)

Figure S10 **Mappability, GC content, and GATA1 motif occurrence of the common and MR-only peaks.** Common refers to common peaks identified by both the MR and the UR samples; MR-only peaks are unique to the MR sample. For the motif occurrence panel, y-axis represents the proportion of peaks with the consensus binding site.
(PDF)

Figure S11 **Mappability scores of GATA1 peaks with respect to 75 mer SETs vs. 36 SETs.** Scatter plot of mappability of GATA1 MR-only peaks with respect to 75 mer versus 36 mer SETs. Shading in the grids represent frequency of data (higher to lower from red to blue). The dark red line is the smooth fit through all the data points.
(PDF)

Figure S12 **Validated GATA1 MR-only peak # 1.** Tag count profiles of the validated MR-only peak with corresponding mappability scores. This peak is within the first intron of the *Zfp637* gene. Peak regions are depicted with black bars.
(PDF)

Figure S13 **Validated GATA1 MR-only peak # 2.** Tag count profiles of the validated MR-only peak with corresponding mappability scores. This peak is within [2 kb, 10 kb] upstream of the transcription start site (TSS) of the *Pb1* gene. Peak regions are depicted with black bars.
(PDF)

Figure S14 **Validated GATA1 MR-only peak # 3.** Tag count profiles of the validated MR-only peak with corresponding mappability scores. This peak is within the first exon of the *Iigp2* gene. Peak regions are depicted with black bars.
(PDF)

Figure S15 **Validated GATA1 MR-only peak # 4.** Tag count profiles of the validated MR-only peak with corresponding mappability scores. This peak is within the sixth intron of the *Polr3h* gene. Peak regions are depicted with black bars.
(PDF)

Figure S16 **Validated GATA1 MR-only peak # 5.** Tag count profiles of the validated MR-only peak with corresponding mappability scores. This peak is within the seventh exon of the *Srsf3* gene. Peak regions are depicted with black bars.
(PDF)

Figure S17 **Validated GATA1 MR-only peak # 6.** Tag count profiles of the validated MR-only peak with corresponding mappability scores. This peak is within [2 kb, 10 kb] upstream of the TSS of *Prim2* gene. Peak regions are depicted with black bars.
(PDF)

Figure S18 **Validated GATA1 MR-only peak # 7.** Tag count profiles of the validated MR-only peak with corresponding mappability scores. This peak is within the first intron of the *Grk5* gene. Peak regions are depicted with black bars.
(PDF)

Figure S19 **Validated GATA1 MR-only peak # 8.** Tag count profiles of the validated MR-only peak with corresponding mappability scores. This peak is within the second intron of the *Ccdc85a* gene. Peak regions are depicted with black bars.
(PDF)

Figure S20 **Scatter plot of average log base 2 ChIP to Input ratio versus ChIP count of GATA1 MR-only peaks.** Green boxes and red circles indicate peaks validated by quantitative real-time ChIP analysis and peaks that are not validated, respectively. Horizontal and vertical lines correspond to average log base 2 ChIP to Input ratio of 2.25 and ChIP count 65, respectively.
(PDF)

Figure S21
**Experimental validation of GATA1 negative peaks.** Quantitative real-time ChIP analysis of sites that were not predicted to be MR peaks, in 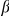-estradiol untreated (−) and 24 hrs treated (+) G1-ER-GATA1 cells based on three independent biological replicates. None of these 7 regions exhibited an increase in GATA1 occupancy in +EST compared to -EST.(PDF)

Table S1 **Summary of number of reads for short and long read datasets.** “(C)” and “(I)” refer to ChIP and input samples, respectively.
(PDF)

Table S2 **Summary of the UR and MR peaks detected by the conditional binomial test.**
(PDF)

Table S3 **Multi-read similarity analysis of STAT1 MR peaks.**
(PDF)

Table S4 **STAT1 MR peaks that share multi-reads with another peak.** In parentheses are the number of peaks classified as Type-II.
(PDF)

Table S5 **Multi-read similarity analysis of GATA1 MR peaks.**
(PDF)

Table S6 **GATA1 MR peaks that share multi-reads with another peak.** In parentheses are the number of peaks classified as Type-II.
(PDF)

Table S7 **Annotation of STAT1 common and MR-only peaks in terms of repeat elements other than segmental duplications.**
(PDF)

Table S8 **Annotation of GATA1 common and MR-only peaks in terms of repeat elements other than segmental duplications.**
(PDF)

Table S9 **Primers used for real-time PCR.**
(PDF)
