# Supplementary figures and images for: Discovering Transcription Factor Binding Sites in Highly Repetitive Regions of Genomes with Multi-Read Analysis of ChIP-Seq Data
Source: PLoS Comput Biol. 2011 Jul 14;7(7):e1002111. doi: 10.1371/journal.pcbi.1002111 (PMC3136429; doi:10.1371/journal.pcbi.1002111)

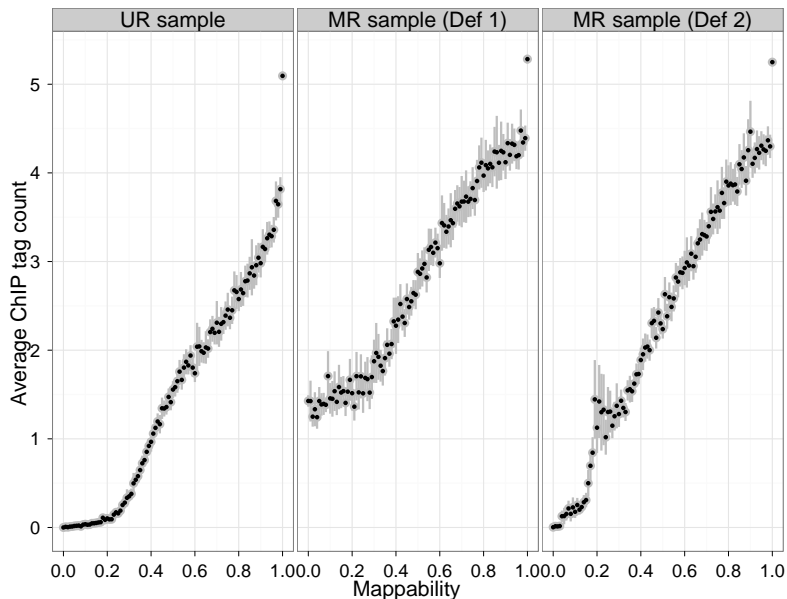

(a) Mappability bias.

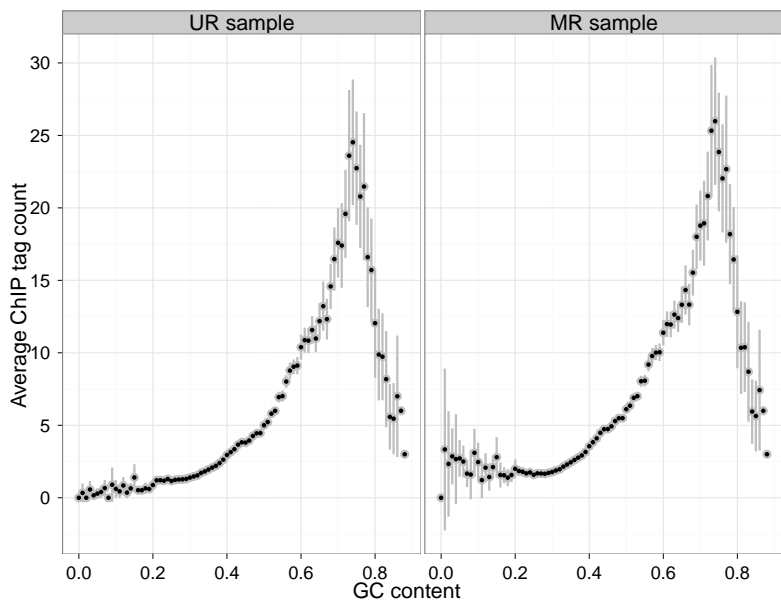

(b) GC content bias.

Supplement: Figure S1 — Mappability and GC content sequence biases in the STAT1 UR and MR samples. Mean tag counts against mappability and GC content in the UR and MR samples, respectively. The patterns observed are typical of ChIP-Seq data with 36 mer to 75 mer tags. “Def 1” and “Def 2” indicate the definitions of mappability for the UR and MR samples, respectively. (PDF) [file pcbi.1002111.s001.pdf]

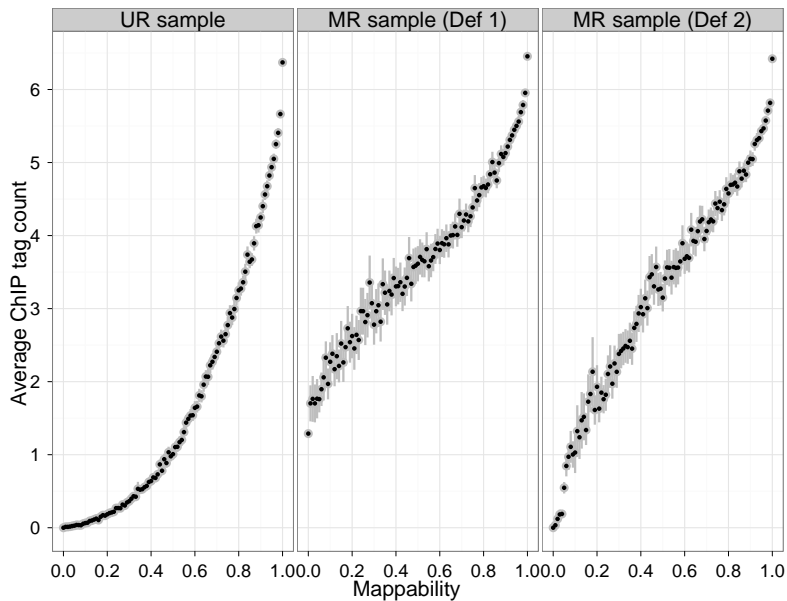

(a) Mappability bias.

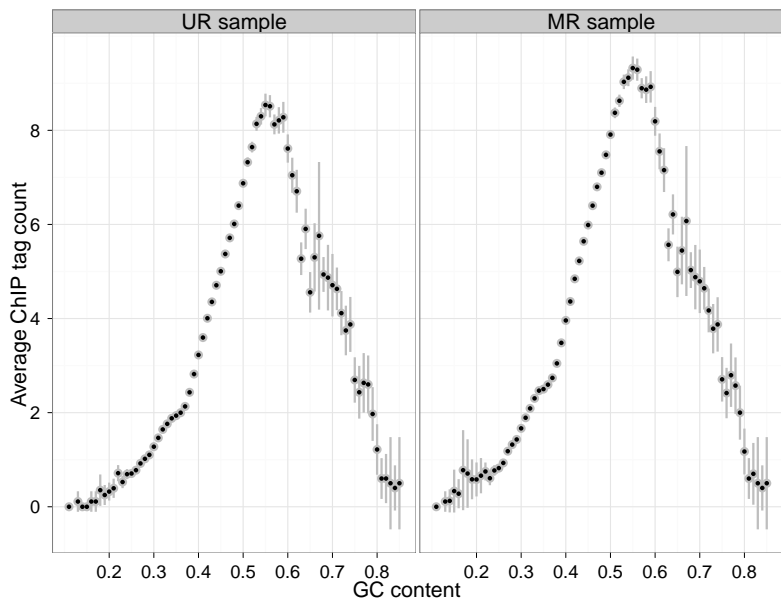

(b) GC content bias.

Supplement: Figure S2 — Mappability and GC content sequences biases in the GATA1 UR and MR samples. Mean tag counts against mappability and GC content in the UR and MR samples, respectively. The patterns observed are typical of ChIP-Seq data with 36 mer to 75 mer tags. “Def 1” and “Def 2” indicate the definitions of mappability for the UR and MR samples, respectively. (PDF) [file pcbi.1002111.s002.pdf]

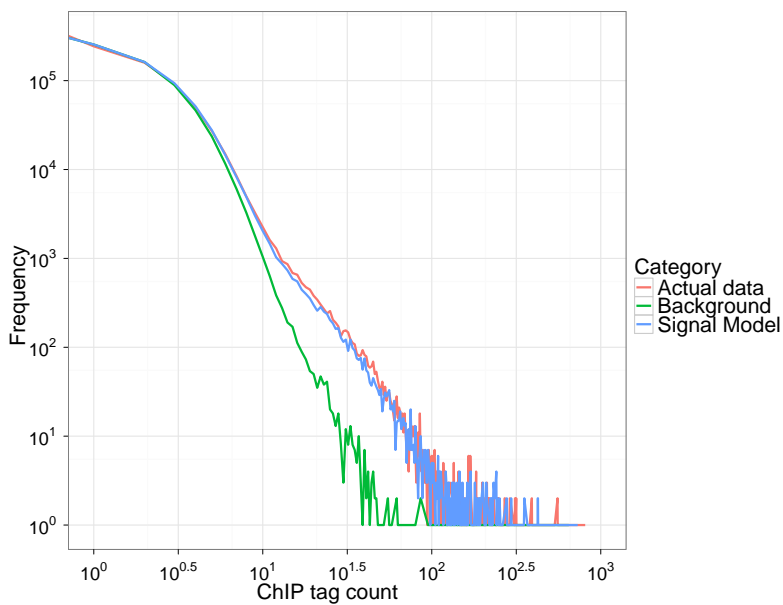

(a) STAT1 UR Sample.

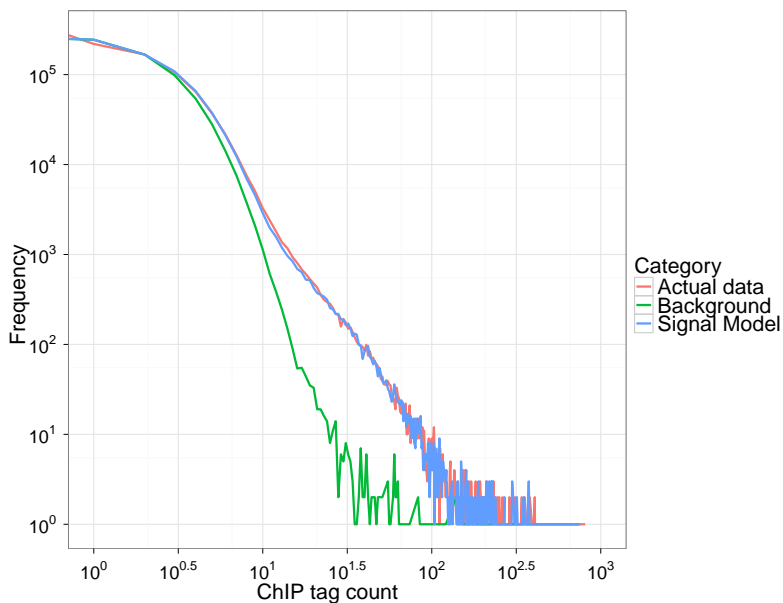

(b) STAT1 MR Sample.

Supplement: Figure S3 — MOSAiCS goodness of fit for the STAT1 UR and MR samples. (a) Goodness of fit for the UR sample. (b) Goodness of fit for the MR sample. Both axes are in log10 scale. (PDF) [file pcbi.1002111.s003.pdf]

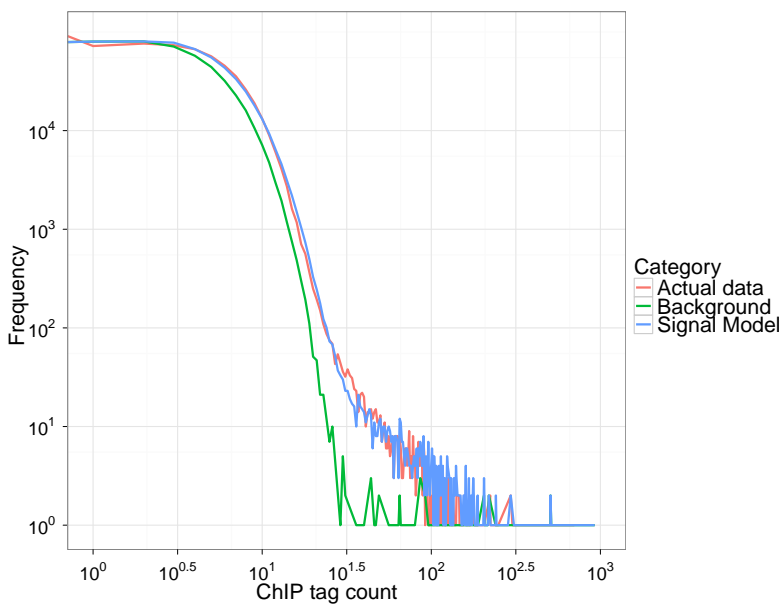

(a) GATA1 UR Sample.

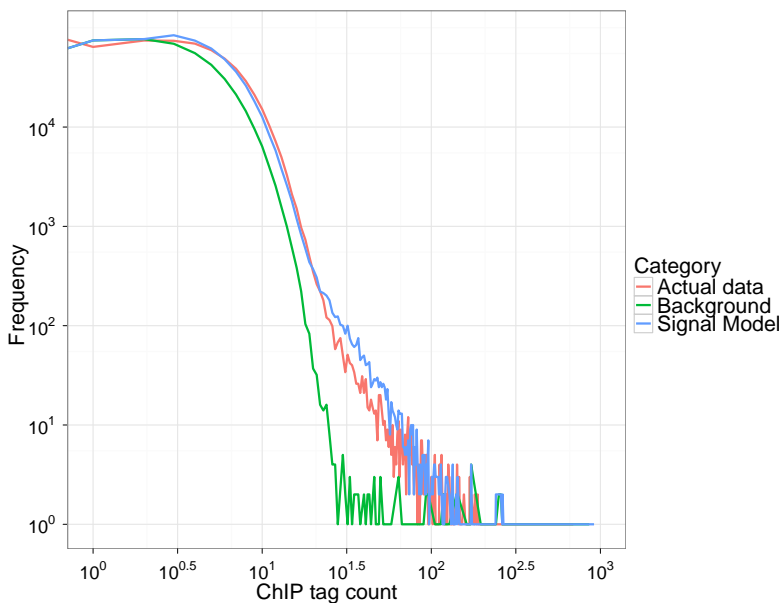

(b) GATA1 MR Sample.

Supplement: Figure S4 — MOSAiCS goodness of fit for the GATA1 UR and MR samples. (a) Goodness of fit for the UR sample. (b) Goodness of fit for the MR sample. Both axes are in log10 scale. (PDF) [file pcbi.1002111.s004.pdf]

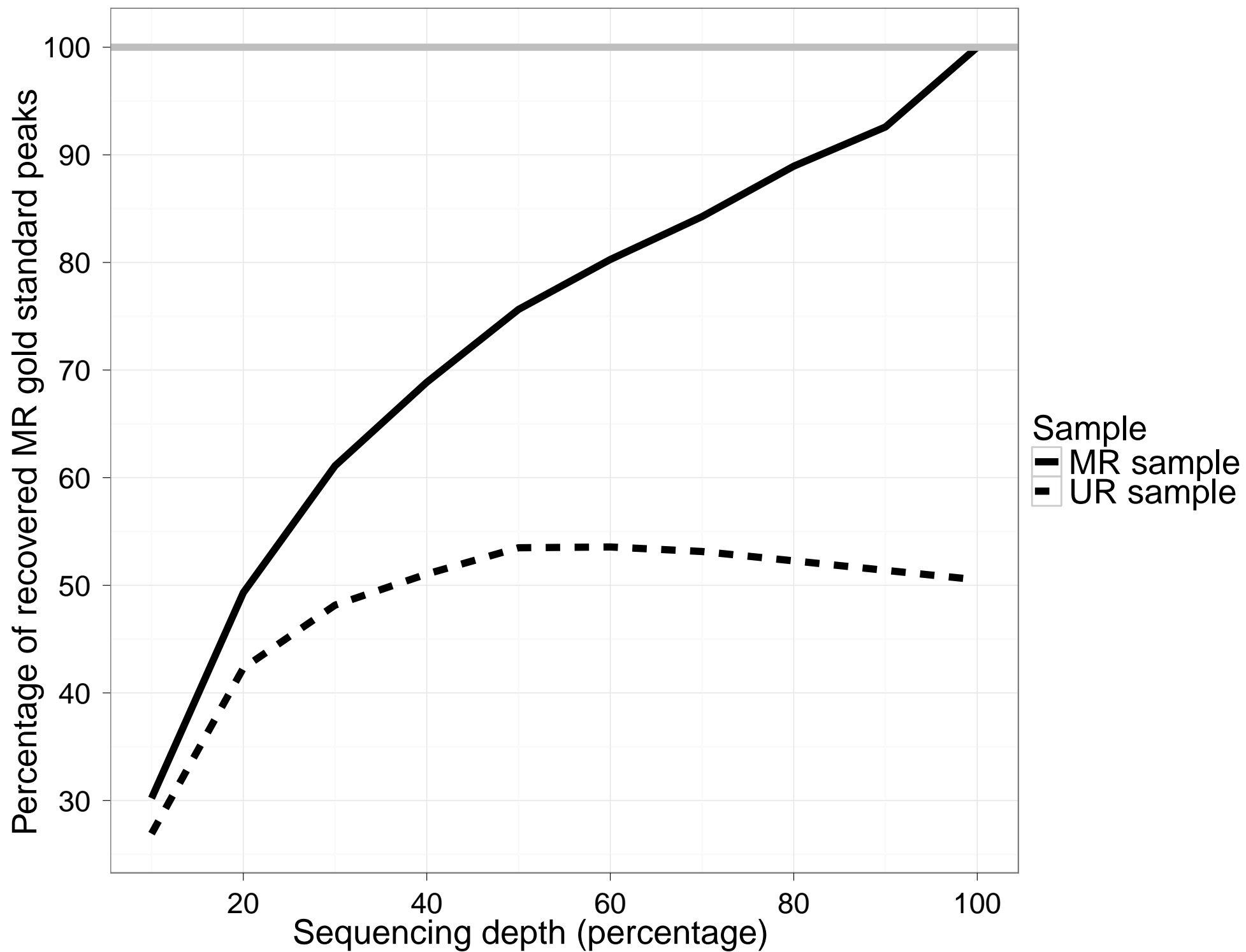

Supplement: Figure S5 — Saturation plot of the STAT1 sample. Percentage of STAT1 MR gold standard peaks recovered by MOSAiCS using sub-sampled UR and MR samples with lower sequencing depths. -axis refers to the percentage of reads sampled from the full dataset. (PDF) [file pcbi.1002111.s005.pdf]

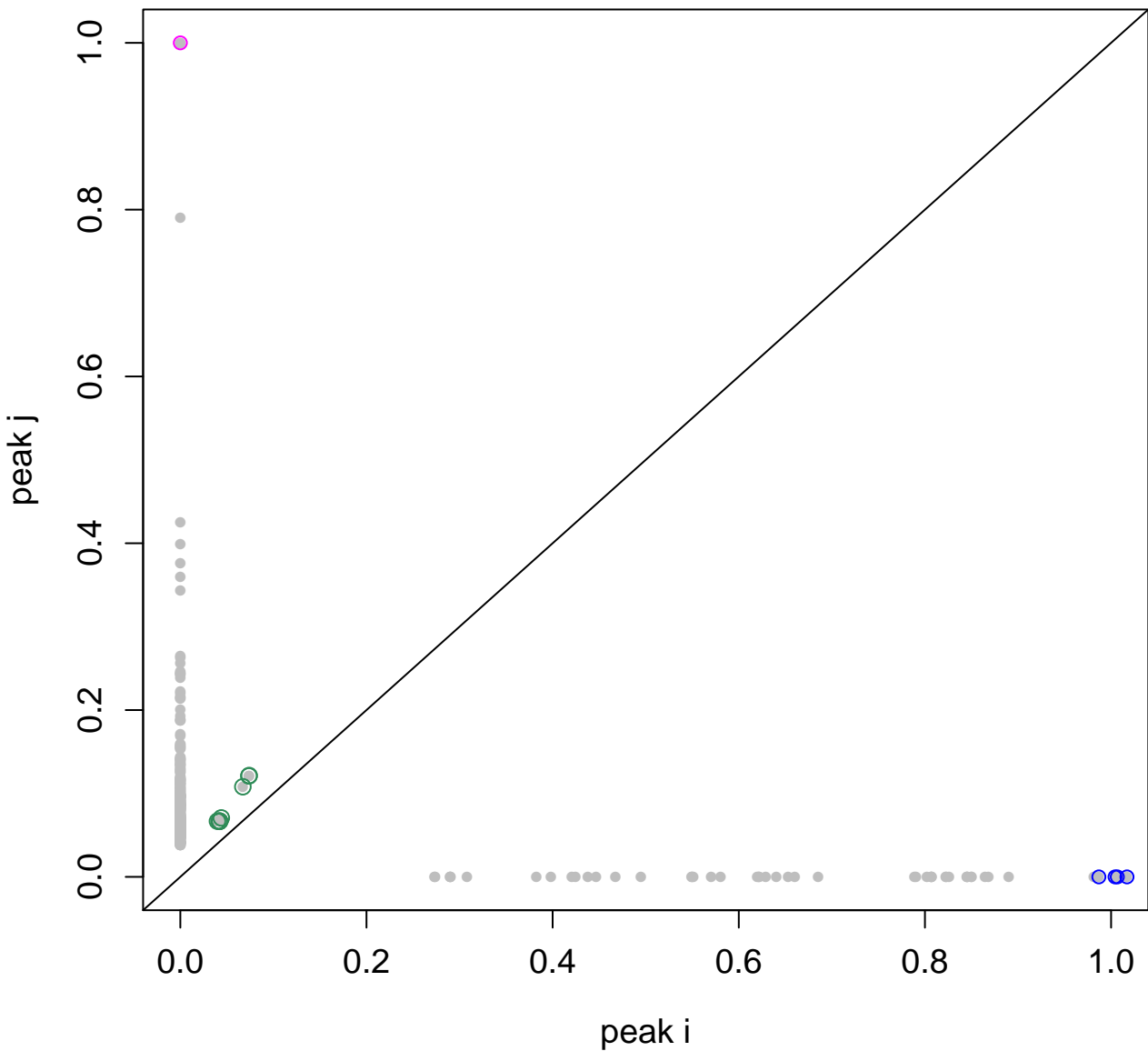

Supplement: Figure S6 — Scatter plot of multi-read weights for a Type-II peak # 1. Peak is a Type-II MR-only peak (chr4: 145,711,000 - 145,711,199) with a total of 54 mapping reads, 4 of which are uni-reads (circled in blue). It shares a maximum of 9 reads (circled in green) with peak with a maximum multi-read similarity of 0.0144. The sums of its fractional counts from unshared and shared multi-reads with peak are 28.25 and 0.47, respectively. Uni-reads of peak are circled in magenta. (PDF) [file pcbi.1002111.s006.pdf]

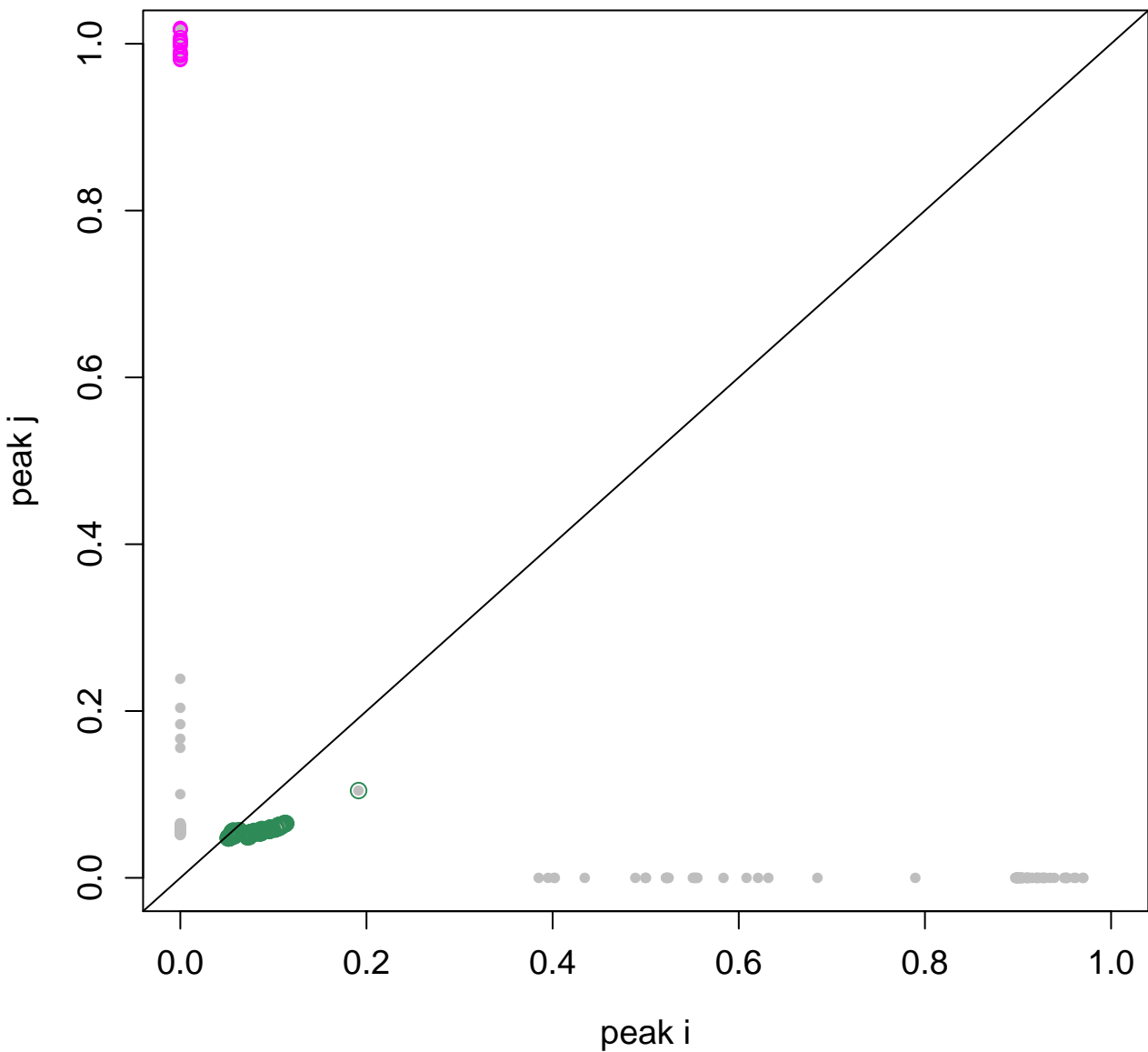

Supplement: Figure S7 — Scatter plot of multi-read weights for a Type-II peak # 2. Peak is a Type-II MR-only peak (chr3: 96,290,200 - 96,290,799) with a total of 328 mapping reads, none of which are uni-reads. It shares a maximum of 236 reads with peak with a maximum multi-read similarity of 0.516. The sums of its fractional counts from unshared and shared multi-reads with peak are 73.94 and 17.30, respectively. Uni-reads of peak are circled in magenta. (PDF) [file pcbi.1002111.s007.pdf]

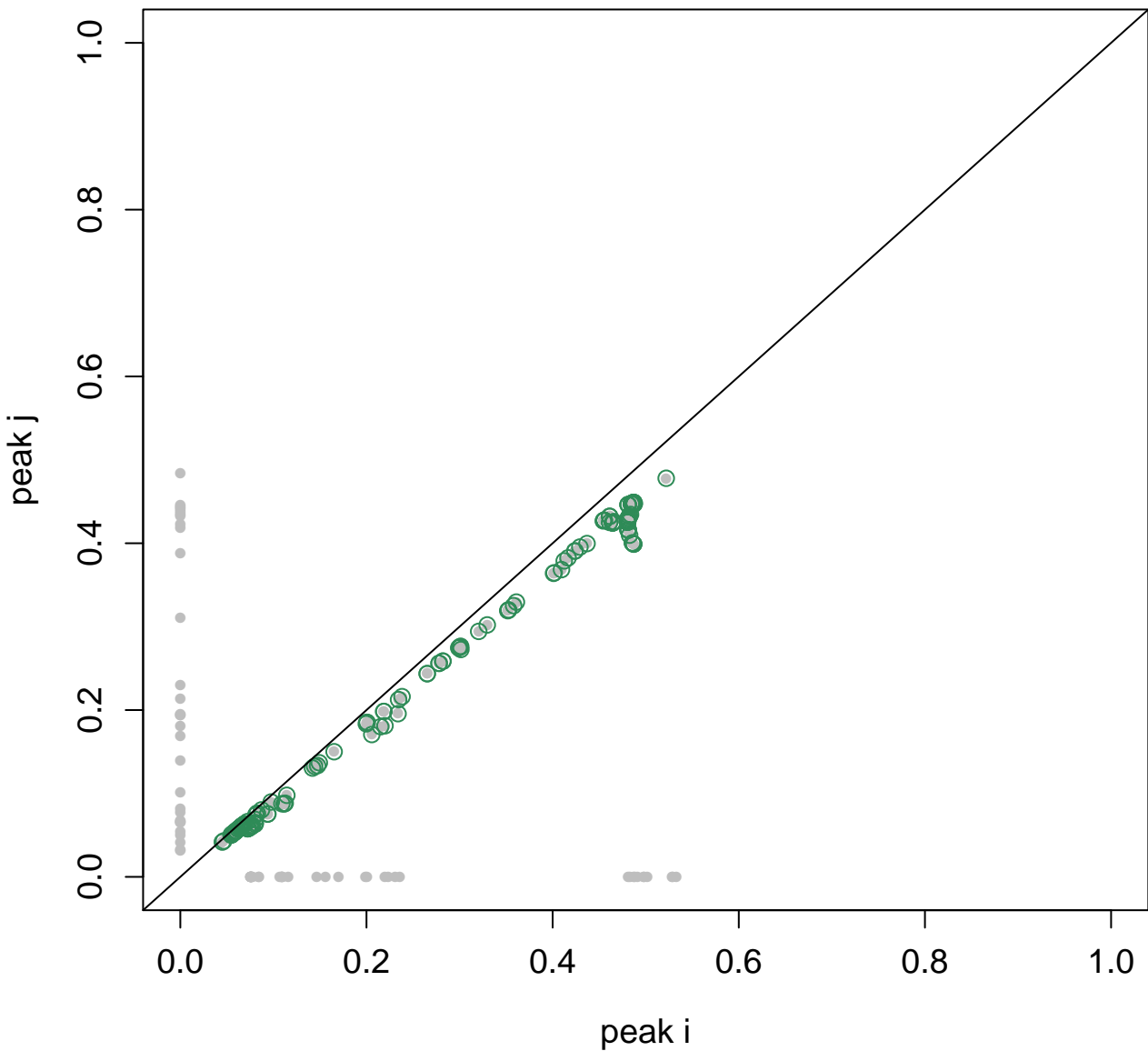

Supplement: Figure S8 — Scatter plot of multi-read weights for a Type-I peak # 1. Peak is a Type-I MR-only peak (chr7: 9,183,800 - 9,184,199) with a total of 200 mapping reads, none of which are uni-reads. It shares a maximum of 161 reads with peak with a maximum multi-read similarity of 0.717. The sums of its fractional counts from unshared and shared multi-reads with peak are 8.97 and 40.45, respectively. (PDF) [file pcbi.1002111.s008.pdf]

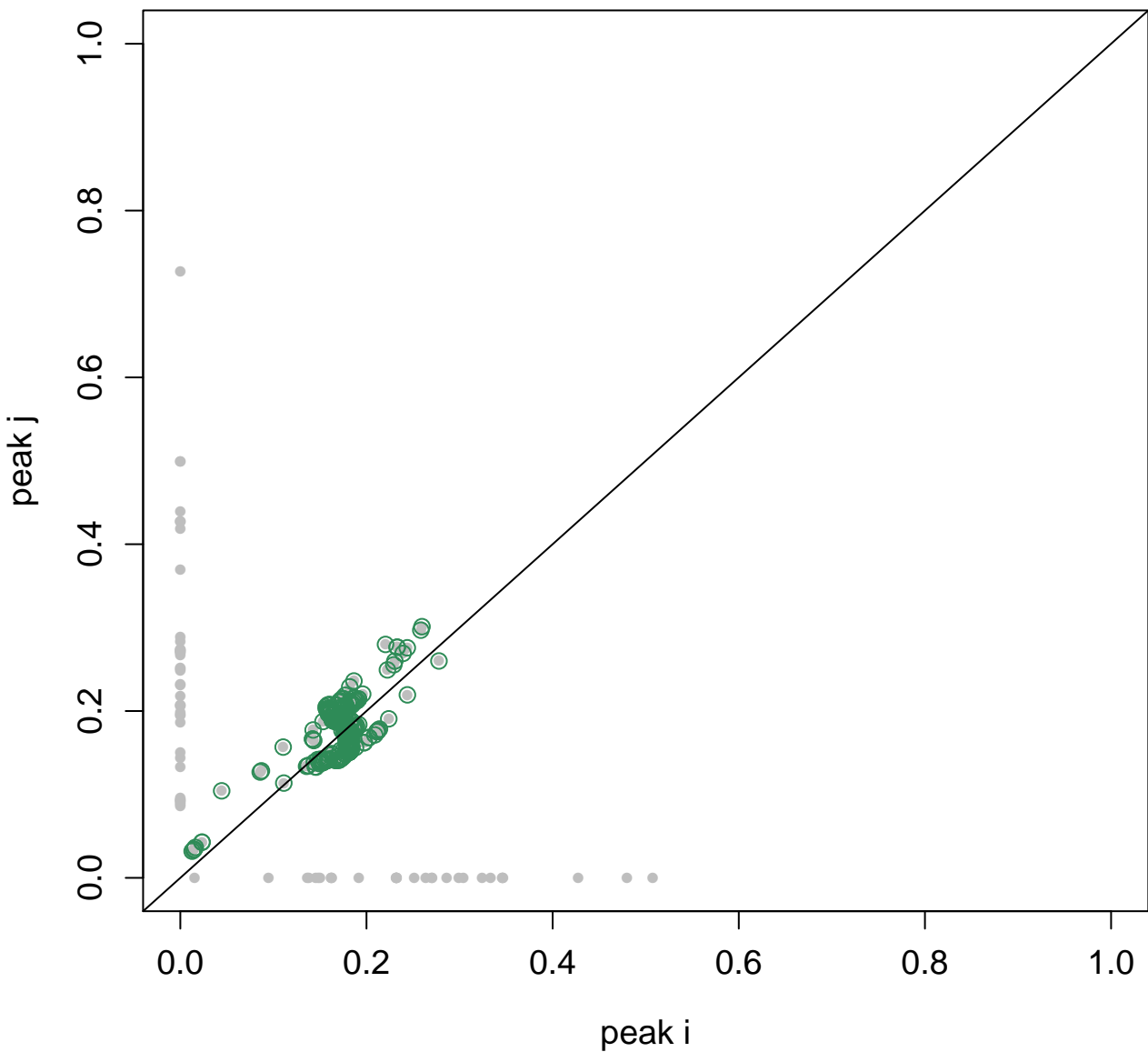

Supplement: Figure S9 — Scatter plot of multi-read weights for a Type-I peak # 2. Peak is a Type-I MR-only peak (chr12: 19,040,000 - 19,040,399) with a total of 343 mapping reads, none of which are uni-reads. It shares a maximum of 312 reads with peak with a maximum multi-read similarity of 0.762. The sums of its fractional counts from unshared and shared multi-reads with peak are 7.73 and 53.68, respectively. (PDF) [file pcbi.1002111.s009.pdf]

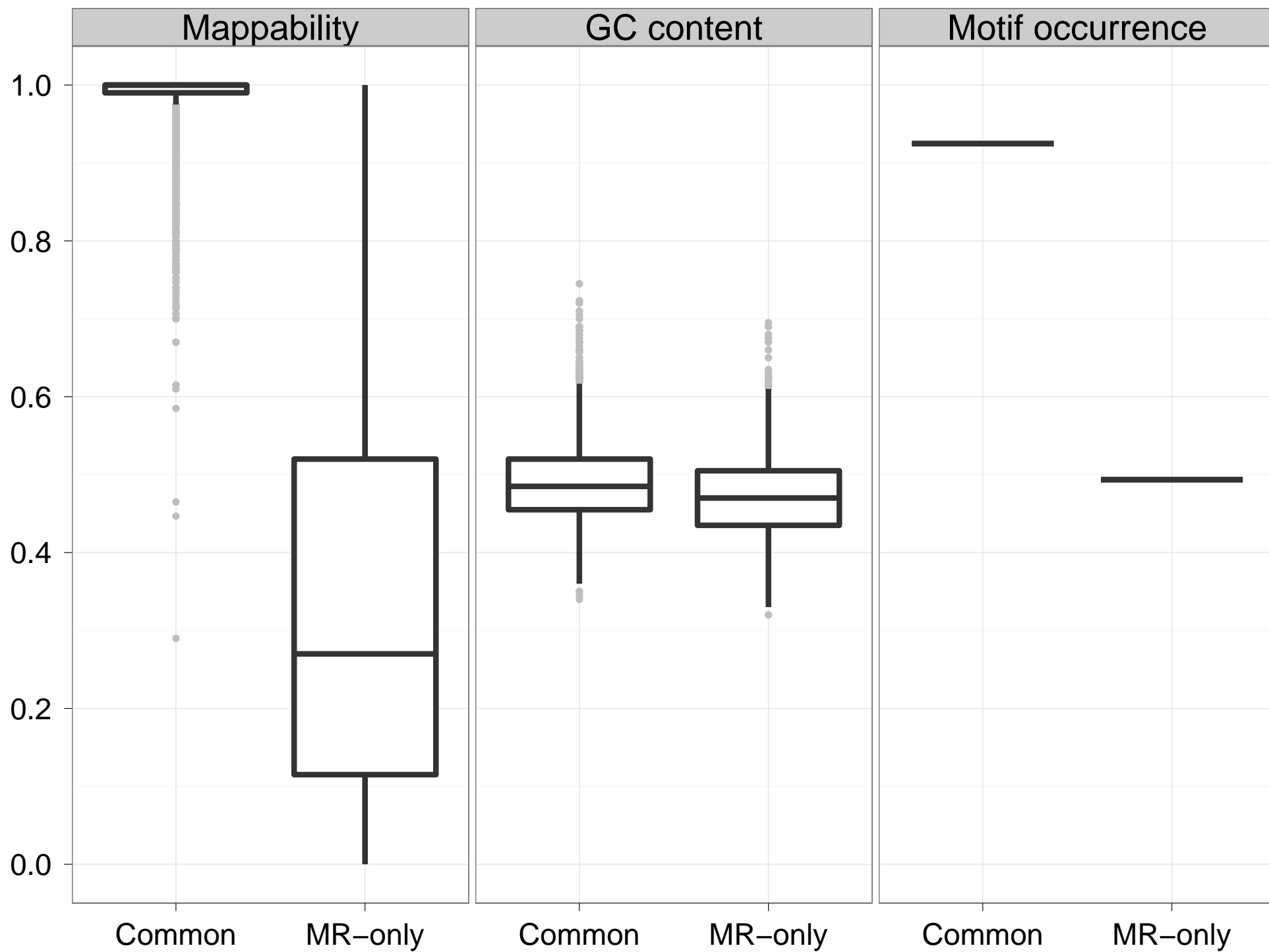

Supplement: Figure S10 — Mappability, GC content, and GATA1 motif occurrence of the common and MR-only peaks. Common refers to common peaks identified by both the MR and the UR samples; MR-only peaks are unique to the MR sample. For the motif occurrence panel, y-axis represents the proportion of peaks with the consensus binding site. (PDF) [file pcbi.1002111.s010.pdf]

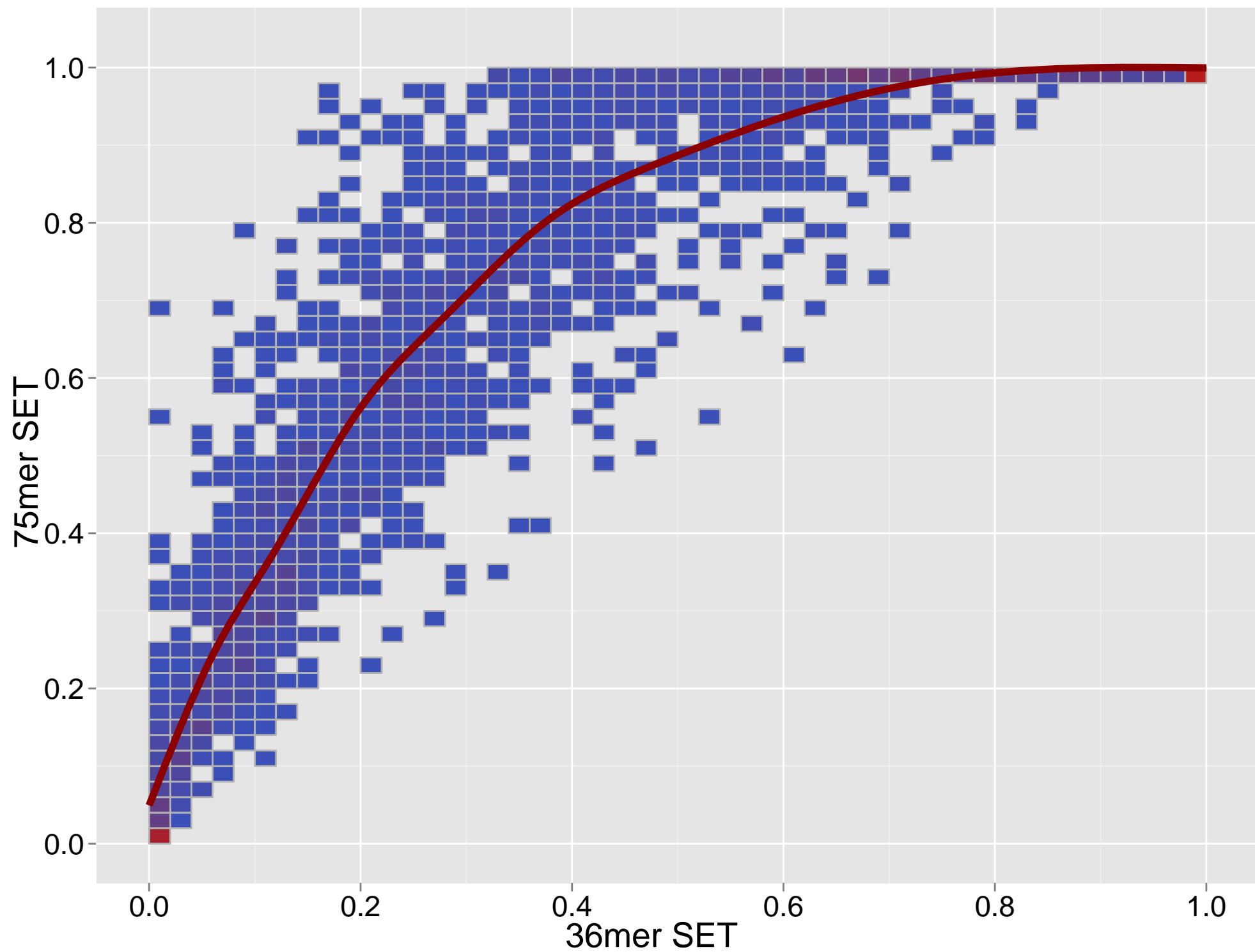

Supplement: Figure S11 — Mappability scores of GATA1 peaks with respect to 75 mer SETs vs. 36 SETs. Scatter plot of mappability of GATA1 MR-only peaks with respect to 75 mer versus 36 mer SETs. Shading in the grids represent frequency of data (higher to lower from red to blue). The dark red line is the smooth fit through all the data points. (PDF) [file pcbi.1002111.s011.pdf]

chr6 : 117794600 – 117794999

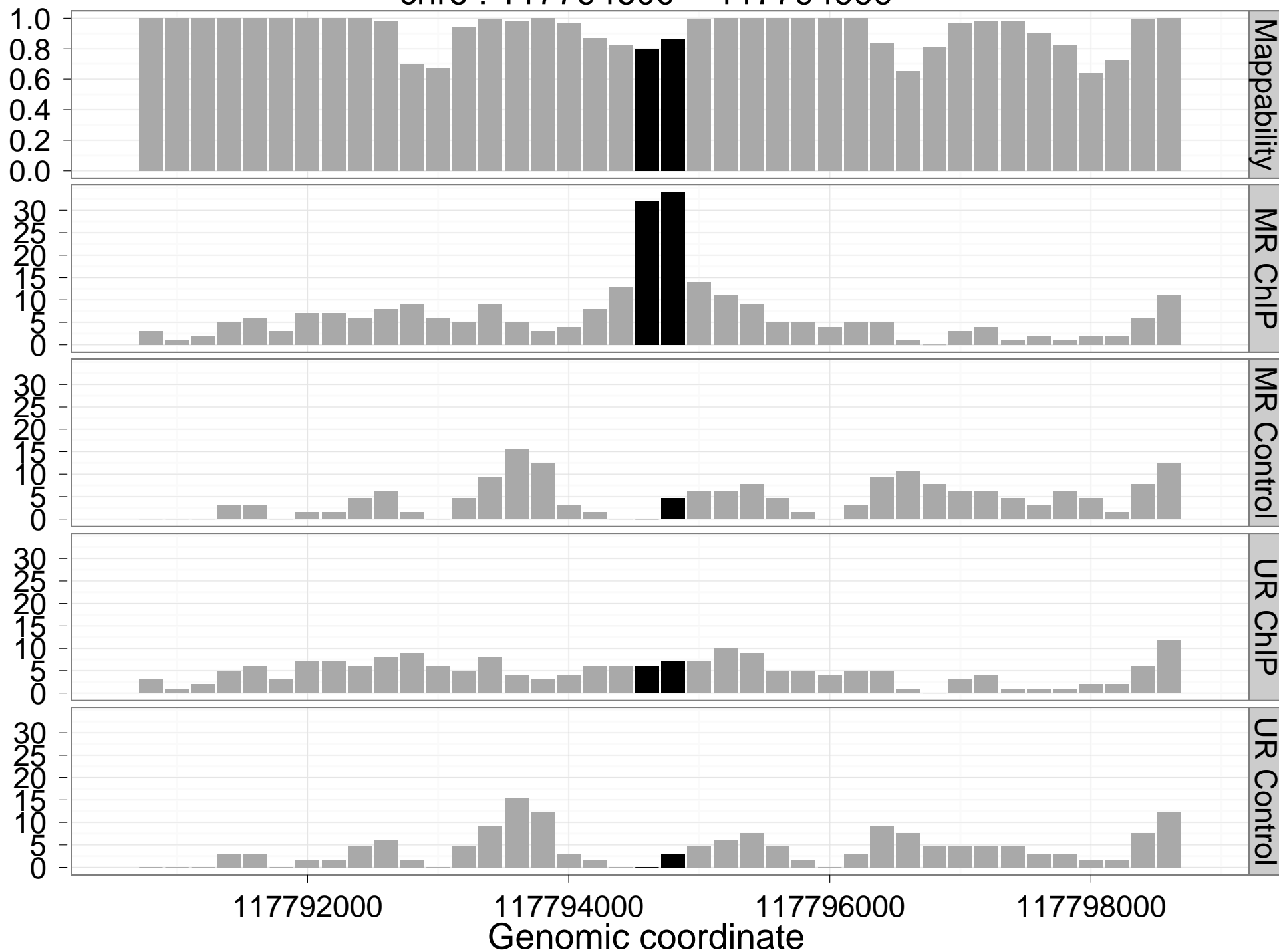

Supplement: Figure S12 — Validated GATA1 MR-only peak # 1. Tag count profiles of the validated MR-only peak with corresponding mappability scores. This peak is within the first intron of the Zfp637 gene. Peak regions are depicted with black bars. (PDF) [file pcbi.1002111.s012.pdf]

chr14 : 31892600 – 31892999

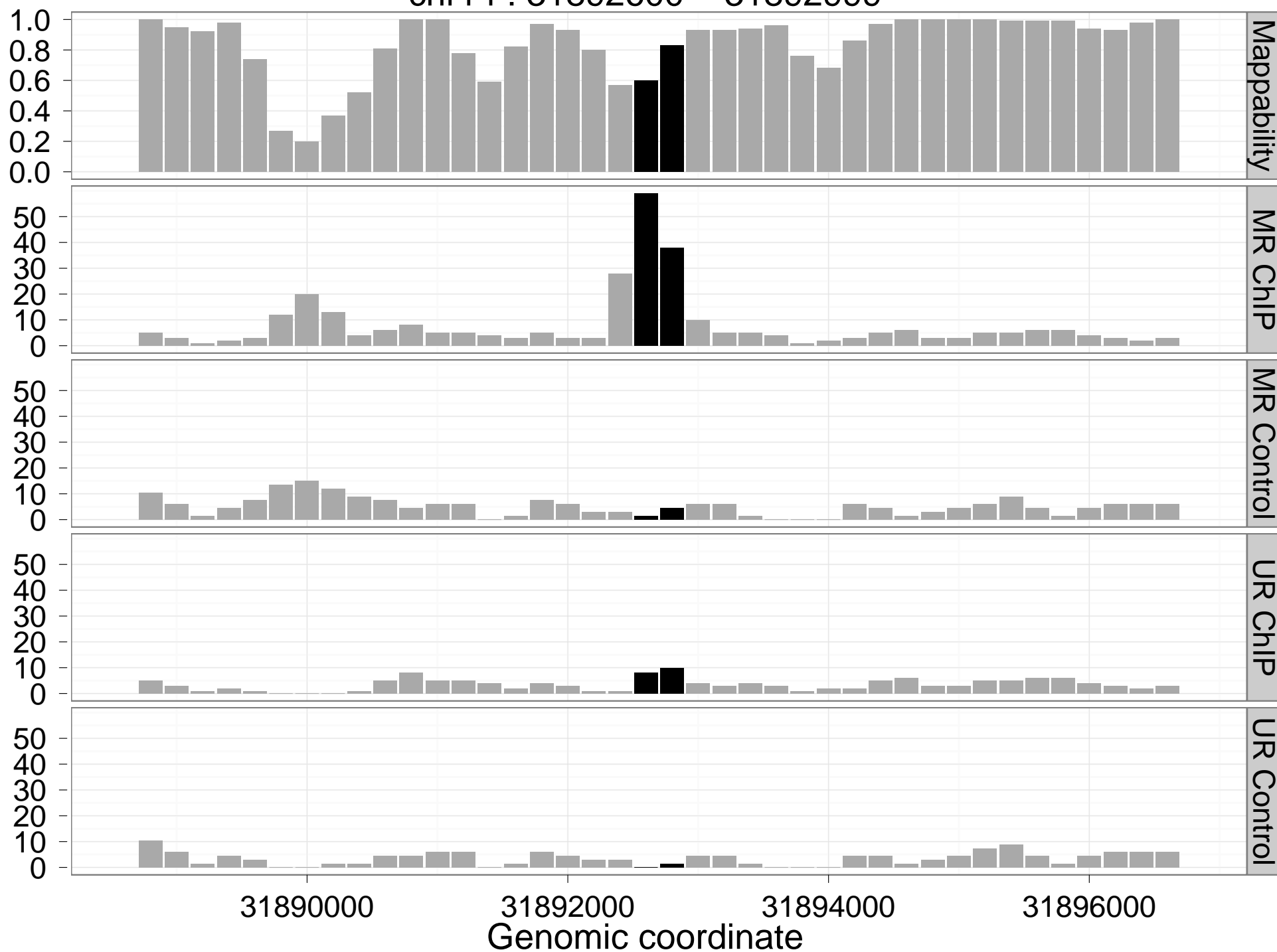

Supplement: Figure S13 — Validated GATA1 MR-only peak # 2. Tag count profiles of the validated MR-only peak with corresponding mappability scores. This peak is within [2 kb, 10 kb] upstream of the transcription start site (TSS) of the Pb1 gene. Peak regions are depicted with black bars. (PDF) [file pcbi.1002111.s013.pdf]

chr11 : 58028200 – 58028799

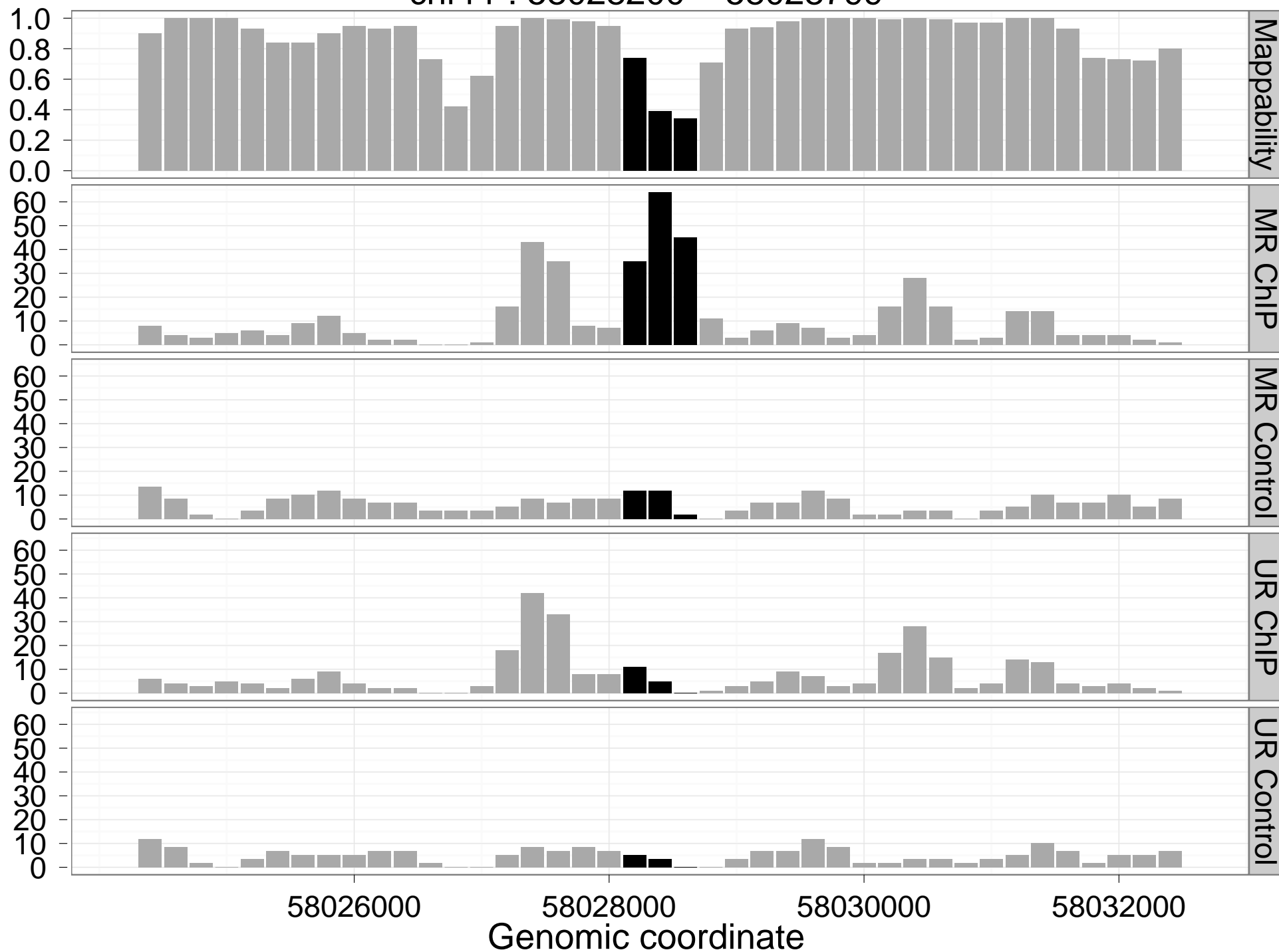

Supplement: Figure S14 — Validated GATA1 MR-only peak # 3. Tag count profiles of the validated MR-only peak with corresponding mappability scores. This peak is within the first exon of the Iigp2 gene. Peak regions are depicted with black bars. (PDF) [file pcbi.1002111.s014.pdf]

chr15 : 81745000 – 81745399

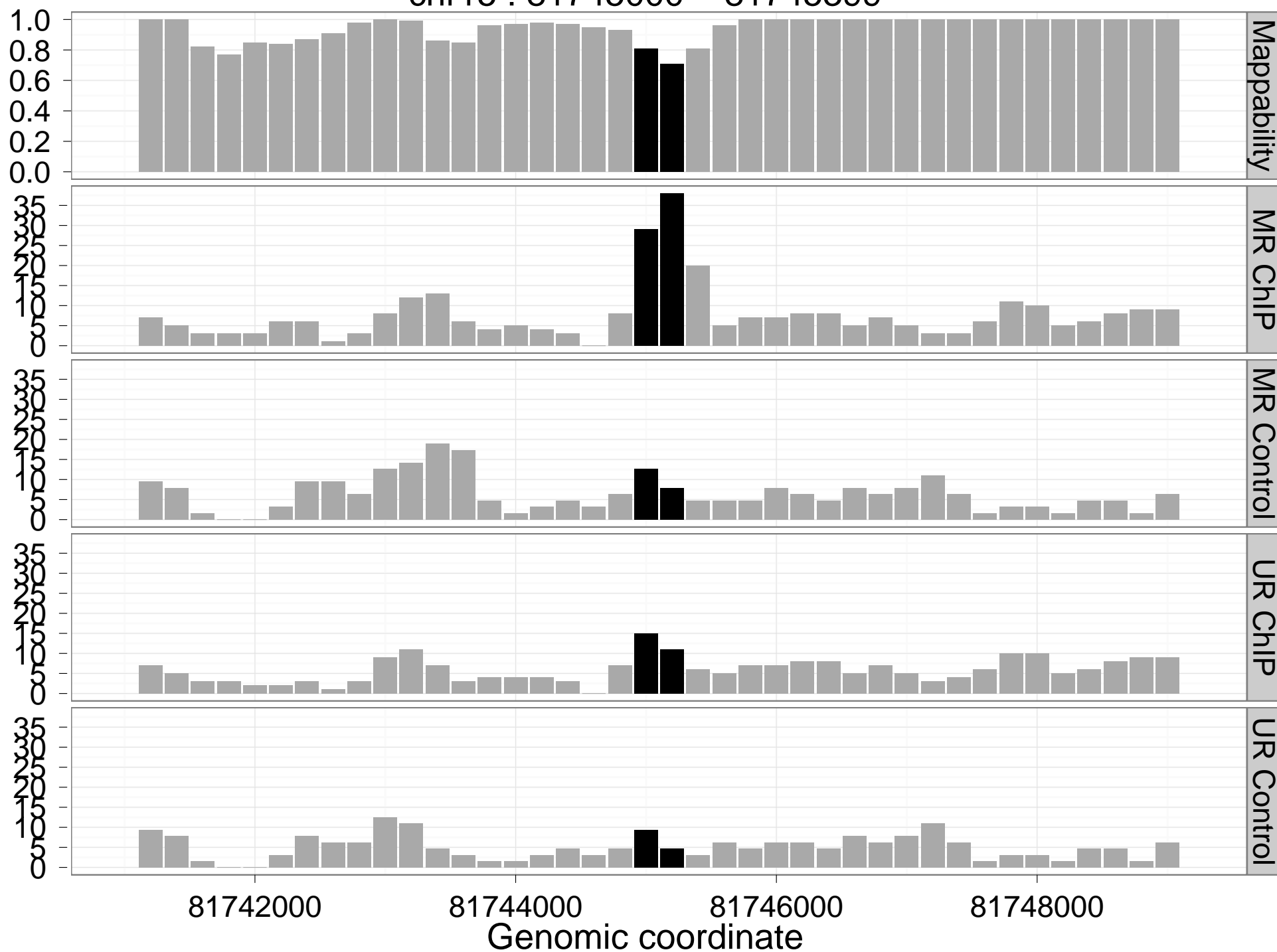

Supplement: Figure S15 — Validated GATA1 MR-only peak # 4. Tag count profiles of the validated MR-only peak with corresponding mappability scores. This peak is within the sixth intron of the Polr3h gene. Peak regions are depicted with black bars. (PDF) [file pcbi.1002111.s015.pdf]

chr17 : 29178600 – 29178799

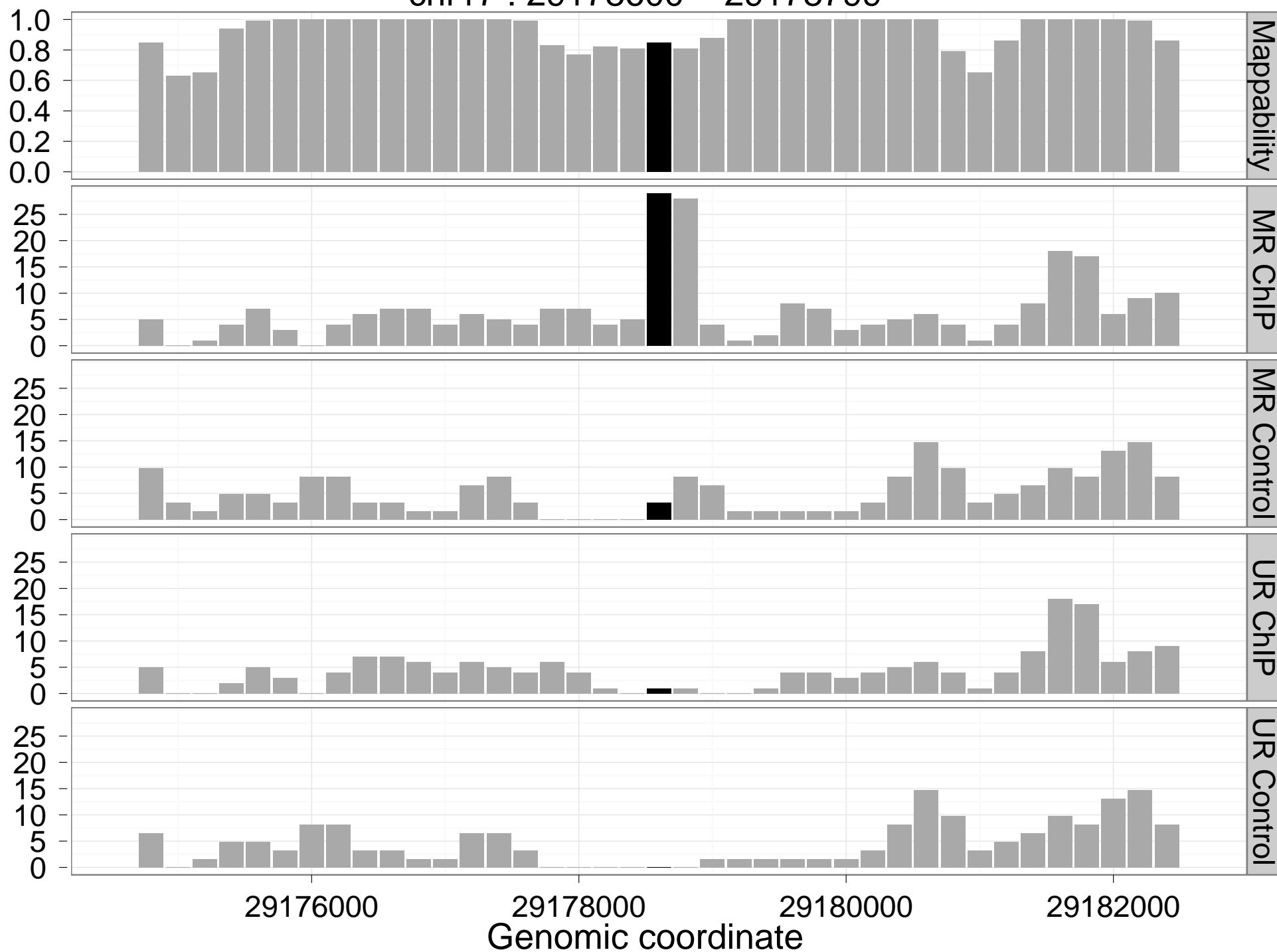

Supplement: Figure S16 — Validated GATA1 MR-only peak # 5. Tag count profiles of the validated MR-only peak with corresponding mappability scores. This peak is within the seventh exon of the Srsf3 gene. Peak regions are depicted with black bars. (PDF) [file pcbi.1002111.s016.pdf]

chr1 : 33687600 – 33688399

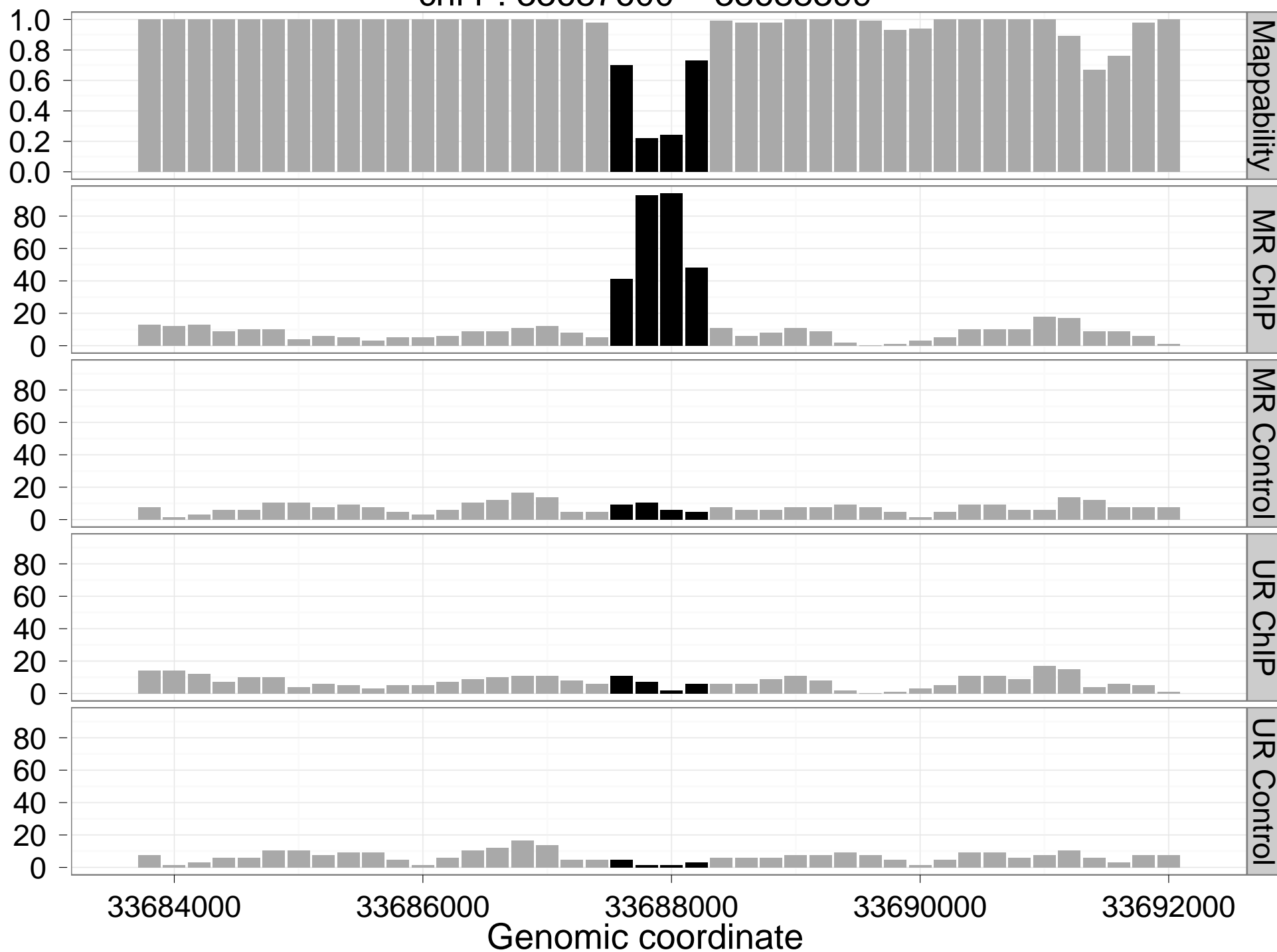

Supplement: Figure S17 — Validated GATA1 MR-only peak # 6. Tag count profiles of the validated MR-only peak with corresponding mappability scores. This peak is within [2 kb, 10 kb] upstream of the TSS of Prim2 gene. Peak regions are depicted with black bars. (PDF) [file pcbi.1002111.s017.pdf]

chr19 : 61000600 – 61000799

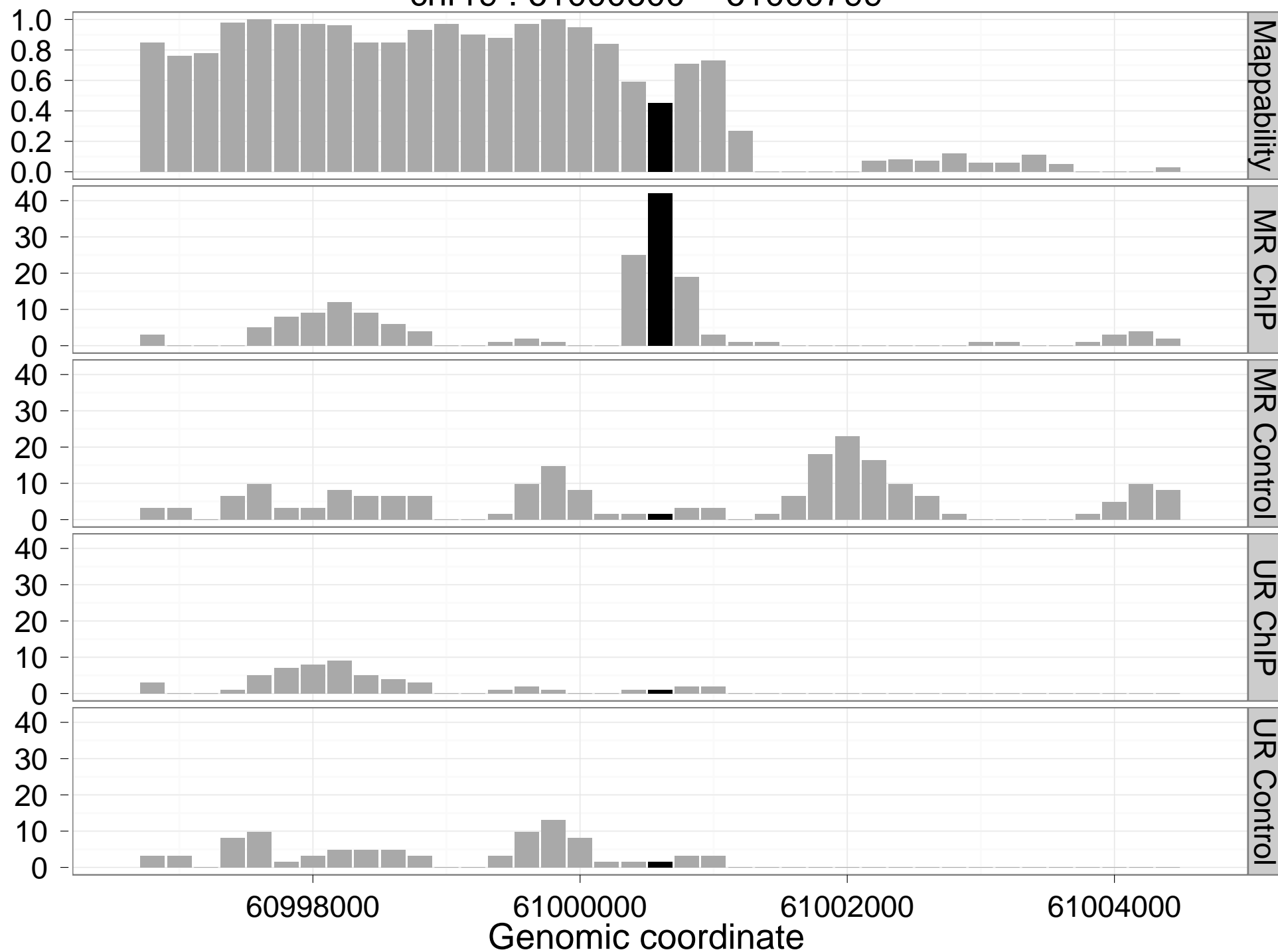

Supplement: Figure S18 — Validated GATA1 MR-only peak # 7. Tag count profiles of the validated MR-only peak with corresponding mappability scores. This peak is within the first intron of the Grk5 gene. Peak regions are depicted with black bars. (PDF) [file pcbi.1002111.s018.pdf]

chr11 : 28404800 – 28405199

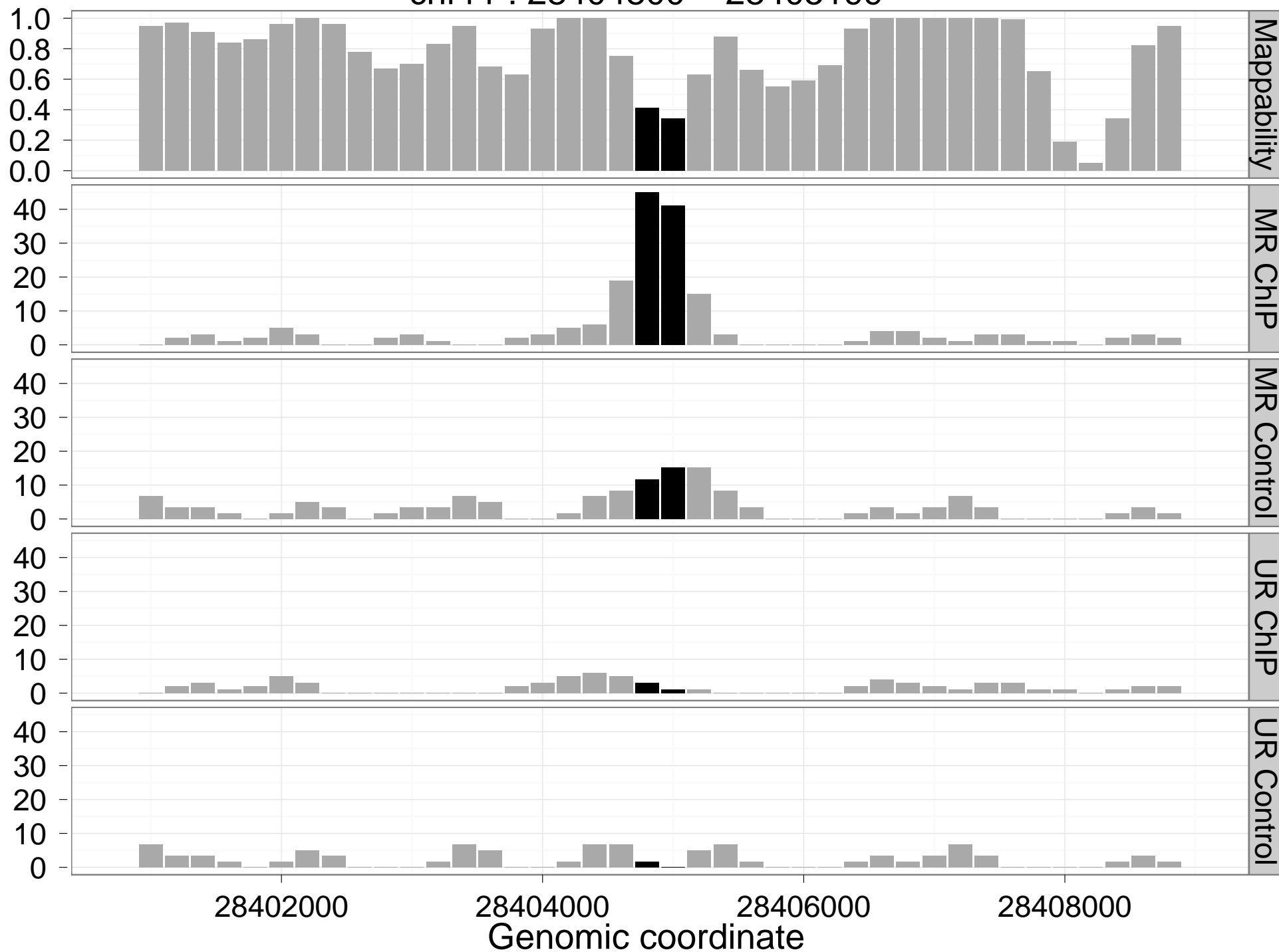

Supplement: Figure S19 — Validated GATA1 MR-only peak # 8. Tag count profiles of the validated MR-only peak with corresponding mappability scores. This peak is within the second intron of the Ccdc85a gene. Peak regions are depicted with black bars. (PDF) [file pcbi.1002111.s019.pdf]

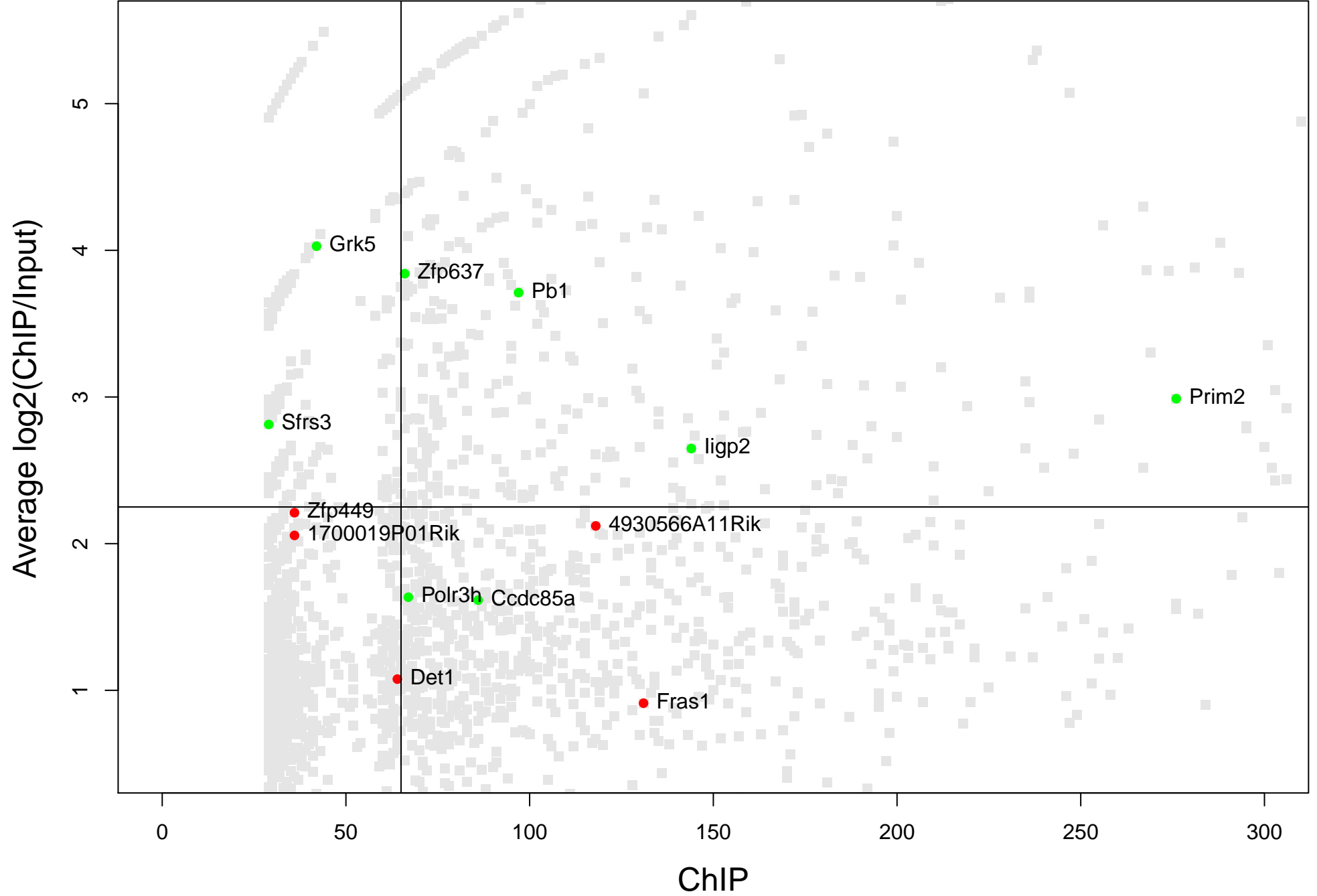

Supplement: Figure S20 — Scatter plot of average log base 2 ChIP to Input ratio versus ChIP count of GATA1 MR-only peaks. Green boxes and red circles indicate peaks validated by quantitative real-time ChIP analysis and peaks that are not validated, respectively. Horizontal and vertical lines correspond to average log base 2 ChIP to Input ratio of 2.25 and ChIP count 65, respectively. (PDF) [file pcbi.1002111.s020.pdf]

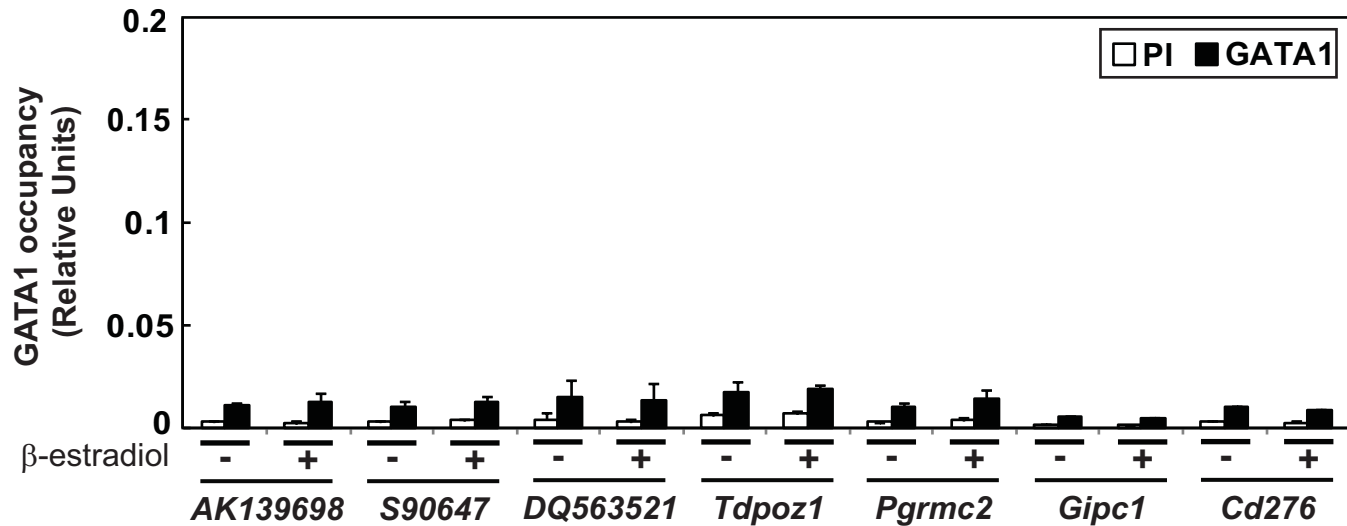

Supplement: Figure S21 — Experimental validation of GATA1 negative peaks. Quantitative real-time ChIP analysis of sites that were not predicted to be MR peaks, in -estradiol untreated (−) and 24 hrs treated (+) G1-ER-GATA1 cells based on three independent biological replicates. None of these 7 regions exhibited an increase in GATA1 occupancy in +EST compared to -EST. (PDF) [file pcbi.1002111.s021.pdf]
