# Supplementary material for: Discovering Transcription Factor Binding Sites in Highly Repetitive Regions of Genomes with Multi-Read Analysis of ChIP-Seq Data
Source: PLoS Comput Biol. 2011 Jul 14;7(7):e1002111. doi: 10.1371/journal.pcbi.1002111 (PMC3136429; doi:10.1371/journal.pcbi.1002111)
Supplement: Table S1 — Summary of number of reads for short and long read datasets. “(C)” and “(I)” refer to ChIP and input samples, respectively. (PDF) [file pcbi.1002111.s022.pdf]

Dataset	# of all reads	# of alignable reads	# of unireads	# of multireads
STAT1(C)	76,913,219	28,180,625	23,011,726	5,168,899
STAT1(I)	49,771,625	23,842,579	19,069,490	4,773,089
GATA1(C)	33,124,216	26,259,170	22,462,757	3,796,413
GATA1(I)	20,711,007	17,060,315	14,369,415	2,690,900
MECP2-SET(C)	15,253,906	12,084,960	9,924,831	2,160,129
MECP2-SET(I)	21,870,009	19,759,268	17,088,510	2,670,758
MECP2-PET(C)	18,622,331	12,765,663	11,963,876	801,787
MECP2-PET(I)	18,498,899	15,586,993	14,598,896	988,097
