# Supplementary material for: Discovering Transcription Factor Binding Sites in Highly Repetitive Regions of Genomes with Multi-Read Analysis of ChIP-Seq Data
Source: PLoS Comput Biol. 2011 Jul 14;7(7):e1002111. doi: 10.1371/journal.pcbi.1002111 (PMC3136429; doi:10.1371/journal.pcbi.1002111)
Supplement: Table S2 — Summary of the UR and MR peaks detected by the conditional binomial test. (PDF) [file pcbi.1002111.s023.pdf]

Dataset	# of UR-only peaks	# of common peaks	# of MR-only peaks
STAT1	0	37233	2965
GATA1	0	6148	2747
