# Supplementary material for: Discovering Transcription Factor Binding Sites in Highly Repetitive Regions of Genomes with Multi-Read Analysis of ChIP-Seq Data
Source: PLoS Comput Biol. 2011 Jul 14;7(7):e1002111. doi: 10.1371/journal.pcbi.1002111 (PMC3136429; doi:10.1371/journal.pcbi.1002111)
Supplement: Table S3 — Multi-read similarity analysis of STAT1 MR peaks. (PDF) [file pcbi.1002111.s024.pdf]

	Common (22420)	MR-only (3255)
No shared multi-reads	12125 (54.08%)	613 (18.83%)
Similarity score > 0.5	108 (0.48%)	564 (17.33%)
Similarity score ≤ 0.5	10187 (45.44%)	2078 (63.84%)
