# Supplementary material for: Discovering Transcription Factor Binding Sites in Highly Repetitive Regions of Genomes with Multi-Read Analysis of ChIP-Seq Data
Source: PLoS Comput Biol. 2011 Jul 14;7(7):e1002111. doi: 10.1371/journal.pcbi.1002111 (PMC3136429; doi:10.1371/journal.pcbi.1002111)
Supplement: Table S5 — Multi-read similarity analysis of GATA1 MR peaks. (PDF) [file pcbi.1002111.s026.pdf]

	Common (6024)	MR-only (2838)
No shared multi-reads	5575 (92.55%)	783 (27.59%)
Similarity score > 0.5	5 (0.08%)	349 (12.30%)
Similarity score ≤ 0.5	444 (7.37%)	1706 (60.11%)
