# Supplementary material for: Discovering Transcription Factor Binding Sites in Highly Repetitive Regions of Genomes with Multi-Read Analysis of ChIP-Seq Data
Source: PLoS Comput Biol. 2011 Jul 14;7(7):e1002111. doi: 10.1371/journal.pcbi.1002111 (PMC3136429; doi:10.1371/journal.pcbi.1002111)
Supplement: Table S6 — GATA1 MR peaks that share multi-reads with another peak. In parentheses are the number of peaks classified as Type-II. (PDF) [file pcbi.1002111.s027.pdf]

	Equal contribution of the weights to the peak pair	Significantly different contribution of the weights to the peak pair
Similarity score > 0.5	130 (11)	219 (33)
Similarity score ≤ 0.5	489 (415)	1216 (904)
