# Supplementary material for: Discovering Transcription Factor Binding Sites in Highly Repetitive Regions of Genomes with Multi-Read Analysis of ChIP-Seq Data
Source: PLoS Comput Biol. 2011 Jul 14;7(7):e1002111. doi: 10.1371/journal.pcbi.1002111 (PMC3136429; doi:10.1371/journal.pcbi.1002111)
Supplement: Table S7 — Annotation of STAT1 common and MR-only peaks in terms of repeat elements other than segmental duplications. (PDF) [file pcbi.1002111.s028.pdf]

All common and MR-only peaks

Repeat Type	Common peaks (22420 peaks)	MR-only peaks (2546 peaks)
DNA	0.100401427	0.071484682
LINE	0.209678858	0.190102121
Low_complexity	0.09014273	0.045168892
LTR	0.145272079	0.33503535
Other	0.000223015	0
RC/Helitron	0.000401427	0
RNA	0.000133809	0.000392773
rRNA	8.92E-05	0.001571092
Satellite	0.000490633	0.04752553
scRNA	0.000356824	0.001178319
Simple_repeat	0.087823372	0.06991359
SINE	0.296253345	0.223095051
snRNA	0.001516503	0.003534957
srpRNA	0.000223015	0
tRNA	0.005396967	0.004713276
Unknown	0.002319358	0.001178319
Unknown?	8.92E-05	0
ANY Repeat Elements	0.641971454	0.754516889

Common and MR-only peaks in NONE category

Repeat Type	Common peaks (8782 peaks)	MR-only peaks (667 peaks)
DNA	0.112275	0.052474
LINE	0.232179	0.202399
Low_complexity	0.047028	0.017991
LTR	0.226144	0.584708
Other	0.000455	0
RC/Helitron	0.000455	0
RNA	0.000228	0
rRNA	0.000228	0.001499
Satellite	0.000342	0.112444
scRNA	0.000569	0.001499
Simple_repeat	0.069346	0.056972
SINE	0.2931	0.158921
snRNA	0.000797	0.004498
srpRNA	0.000342	0
tRNA	0.008426	0
Unknown	0.002847	0
Unknown?	0.000114	0
ANY Repeat Elements	0.673423	0.953523
