# Supplementary material for: Discovering Transcription Factor Binding Sites in Highly Repetitive Regions of Genomes with Multi-Read Analysis of ChIP-Seq Data
Source: PLoS Comput Biol. 2011 Jul 14;7(7):e1002111. doi: 10.1371/journal.pcbi.1002111 (PMC3136429; doi:10.1371/journal.pcbi.1002111)
Supplement: Table S8 — Annotation of GATA1 common and MR-only peaks in terms of repeat elements other than segmental duplications. (PDF) [file pcbi.1002111.s029.pdf]

All common and MR-only peaks

Repeat Type	Common peaks(6024 peaks)	MR-only peaks (2146 peaks)
DNA	0.027722	0.014911
LINE	0.083997	0.190121
Low_complexity	0.028718	0.025629
LTR	0.108898	0.342032
Other	0.004316	0.00699
RC/Helitron	0.000166	0
RNA	0.000996	0
rRNA	0.000664	0.003728
Satellite	0.001328	0.002796
scRNA	0.001494	0.001398
Simple_repeat	0.101262	0.094129
SINE	0.258134	0.140727
snRNA	0.000664	0.003728
srpRNA	0.000166	0.001398
tRNA	0.002656	0
Unknown	0.001826	0.001864
Unknown/Y-chromosome	0.000166	0
ANY Repeat Elements	0.48755	0.658434

Common and MR-only peaks in NONE category

Repeat Type	Common peaks (1347 peaks)	MR-only peaks (526 peaks)
DNA	0.03118	0.003802
LINE	0.092799	0.226236
Low_complexity	0.03712	0.011407
LTR	0.164811	0.456274
Other	0.008909	0.011407
RC/Helitron	0	0
RNA	0.000742	0
rRNA	0.001485	0.005703
Satellite	0.000742	0.001901
scRNA	0.001485	0
Simple_repeat	0.124722	0.072243
SINE	0.242762	0.074144
snRNA	0.000742	0.005703
srpRNA	0	0
tRNA	0.002227	0
Unknown	0.00297	0
Unknown/Y-chromosome	0	0
ANY Repeat Elements	0.539718	0.764259
