# Supplementary material for: Discovering Transcription Factor Binding Sites in Highly Repetitive Regions of Genomes with Multi-Read Analysis of ChIP-Seq Data
Source: PLoS Comput Biol. 2011 Jul 14;7(7):e1002111. doi: 10.1371/journal.pcbi.1002111 (PMC3136429; doi:10.1371/journal.pcbi.1002111)
Supplement: Table S9 — Primers used for real-time PCR. (PDF) [file pcbi.1002111.s030.pdf]

Sl No	Name	Peak position	Primer sequence (5’-3’)
1	Pb1 Forward	Chr14:31892400:31892999	GTGGTCTTGAATTTCTTTTGGATTC
2	Pb1 Reverse	Chr14:31892400:31892999	TCAATGGACCCAAATCCCC
3	Srsf3 Forward	Chr17:29178600:29178999	ATAAACCTTAGGTCCTGCCCAGT
4	Srsf3 Reverse	Chr17:29178600:29178999	GTGTCTCAGCTACTAGAGGACTATAGCTTC
5	Iigp2 Forward	Chr11:58028200:58028999	TCGGAGTGCCTGGTGTCTG
6	Iigp2 Reverse	Chr11:58028200:58028999	AAGACCCACAAACCCAGGG
7	Polr3h Forward	Chr15:81745000:81745599	AAGATGGTGACCAGACCTGCTT
8	Polr3h Reverse	Chr15:81745000:81745599	CAAACTGAACAGGAAGGCGG
9	Zfp637 Forward	Chr6:117794600:117794999	GTGATCGCCCCATGCTCCCA
10	Zfp637 Reverse	Chr6:117794600:117794999	GGCTTCTGATGTGCCGGGCAT
11	Prim2 Forward	Chr1:33687600:33688599	TTTTATCCGGTAAAGCGAATGATTAG
12	Prim2 Reverse	Chr1:33687600:33688599	TCCATGCCAGCGAGCC
13	Ccdc85a Forward	Chr11:28404600:28405199	AGGTGGTTAGCGATTTGTGGA
14	Ccdc85a Reverse	Chr11:28404600:28405199	TGACTTTCCTCCTTACACAAAATCAC
15	Grk5 Forward	Chr19:61000400:61000999	AACCTGGTCTACAGAATGAGGACTG
16	Grk5 Reverse	Chr19:61000400:61000999	CCAAAGGACTTCTGAATACCATACTATG

17	AK139698 Forward	Chr14:54270800-54271199	ACAGCGATTAGCACGTGAAGTG
18	AK139698 Reverse	Chr14:54270800-54271199	TCTCTCCTGACTAGCTGTGGTCTG
19	S90647 Forward	Chr13: 68481800- 68482199	TGAGGGAGGAAATGGCGAG
20	S90647 Reverse	Chr13: 68481800- 68482199	GGCCCAGCACCTCCCTAT
21	DQ563521 Forward	Chr8:119753600- 119753999	AGAAATATGCCTGCCTTTACCTCC
22	DQ563521 Reverse	Chr8:119753600- 119753999	AAA AGATAGGGAAAGCCGGG
23	Tdpoz1 Forward	Chr3: 93980800- 93981199	TTGTGCTGTATTTTTTTTGCCA
24	Tdpoz1 Reverse	Chr3: 93980800- 93981199	CAGGACTGTTTAGATGATTTGCAGG
25	Cd276 Forward	Chr9:58370800:58371399	GTAGAAACTATATCCCGCCCGA
26	Cd276 Reverse	Chr9:58370800:58371399	TTCTTTAGATAGGAGCCCTTCTGG
27	Gipc1 Forward	Chr8:86172000- 86172399	CCAGGGAGTGGCACTATTTGA
28	Gipc1 Reverse	Chr8:86172000- 86172399	GAGACATTGGGCCTGGCAT
29	Pgrmc2 Forward	Chr3: 40868200-40868799	AACTTGTATCTCCCAGCTGCAAG
30	Pgrmc2 Reverse	Chr3: 40868200-40868799	CACCTGCAATGGCTATTCCTG
